# Time-Resolved Transcriptomic and Widely Targeted Metabolomic Profiling of Tomato Seedlings During Short-Term Chilling

**DOI:** 10.3390/biom16101390

**Published:** 2026-09-23

**Authors:** Dan Zhou, Zijun Yan, Tao Yan, Xiumei Fan, Zijuan Xu, Tianhong Wang, Xiaocui Chen, Xiaosong Gou, Sheng Sun, Zhifeng Chen

**Affiliations:** 1 College of Horticulture, Shanxi Agricultural University, Jinzhong 030801, China; 18404968223@163.com (D.Z.);; 2 Zunyi Academy of Agricultural Sciences, Zunyi 563000, China; 3 College of Ecology, Zunyi Normal University, Zunyi 563006, China

**Keywords:** tomato, chilling stress, time-course analysis, transcriptomics, widely targeted metabolomics, phenylpropanoid biosynthesis

## Abstract

(1) Background: Chilling is a major environmental constraint on tomato growth and development, yet the temporal coordination of physiological, transcriptional, and metabolic responses remains insufficiently characterized. (2) Methods: Tomato seedlings were subjected to 4 °C chilling treatment for 0, 1, 3, 6, 12, and 24 h. We measured antioxidant enzyme activities and malondialdehyde (MDA) content, and performed transcriptomic and widely targeted metabolomic analyses to dissect time-course responses. qRT-PCR was used to validate gene-expression profiles. (3) Results: Superoxide dismutase (SOD) and catalase (CAT) activities, together with MDA content, were rapidly altered at early chilling stages, while peroxidase (POD) activity markedly accumulated at 12 and 24 h, showing divergent physiological response patterns. In contrast to the 0 h control, 4819, 4471, 4442, 5742, and 8701 differentially expressed genes (DEGs) were detected at 1, 3, 6, 12, and 24 h, among which 945 DEGs were common across all time-point comparisons. The pronounced rise in DEG count at 24 h reflected large-scale late-stage transcriptional reprogramming. Correspondingly, 262, 173, 170, 247, and 195 differentially abundant metabolites (DAMs) were identified, with merely 26 shared DAMs across all five time points and the maximum DAM number occurring at 1 h, demonstrating fast but non-monotonic metabolic shifts. A pathway-level comparison pinpointed carbon metabolism, phenylpropanoid biosynthesis, and tyrosine metabolism as key perturbed pathways. qRT-PCR results generally validated the RNA-seq expression trends of eight selected genes and showed a significant positive correlation with RNA-Seq data (*r* = 0.852, *p* < 0.01). (4) Conclusions: This study demonstrates stage-dependent and partially asynchronous physiological, transcriptional, and metabolic responses upon short-term chilling in tomato seedlings. Candidate pathways, genes, and metabolites uncovered herein provide valuable resources for further functional characterization of tomato cold responses.

## 1. Introduction

Tomato (*Solanum lycopersicum* L.) is a thermophilic crop that is highly sensitive to chilling temperatures [1]. In protected tomato production, particularly during overwinter cultivation, plants may frequently experience suboptimal temperatures that restrict normal growth and productivity [1]. Chilling can impair photosynthetic performance, membrane stability, cellular metabolism, and redox homeostasis, thereby affecting plant growth and development [2]. Understanding how tomato responds to low temperature is therefore important for improving crop management and developing strategies for cold-resilient production.

Plants respond to low temperature through coordinated physiological and molecular adjustments. The C-repeat binding factor (CBF) pathway is a key component of plant cold signaling and regulates numerous cold-responsive genes [3]. Regulators associated with the ICE–CBF–COR signaling module further contribute to transcriptional responses triggered by low temperature [4]. Chilling also perturbs cellular redox homeostasis, and antioxidant systems play important roles in physiological adjustment to environmental stress [5]. These responses are dynamic, suggesting that different physiological and molecular processes may predominate at different stages of chilling exposure.

Several cold-responsive genes have been functionally characterized in tomato. *SlCOR413IM1* and *SlICE1* have been shown to contribute to chilling tolerance in tomato [6,7]. *SlGRAS4* regulates multiple stress-responsive processes associated with chilling tolerance [8]. The ethylene response factor *SlERF.B8* also contributes to tomato cold tolerance by promoting jasmonate biosynthesis [9]. Although these studies have advanced our understanding of individual regulatory components, cold responses involve extensive changes across transcriptional and metabolic networks that cannot be fully captured by analyses of individual genes.

Integrated transcriptomic and metabolomic analyses enable molecular responses to be examined across multiple regulatory layers. In tomato fruit, this approach has revealed extensive transcriptomic and metabolic changes associated with chilling injury [10]. Similar studies have identified cold-responsive genes, metabolites, and metabolic pathways in pepper [11] and peach [12]. However, the temporal relationships among physiological, transcriptional, and metabolic adjustments during short-term chilling in tomato seedlings remain poorly resolved.

In this study, tomato seedlings were exposed to 4 °C for 0, 1, 3, 6, 12, and 24 h. We combined physiological measurements, RNA sequencing, and widely targeted metabolomic profiling to characterize time-dependent responses to chilling. Specifically, we aimed to (i) determine the temporal patterns of antioxidant enzyme activities and MDA content, (ii) characterize dynamic changes in gene expression and metabolite abundance, and (iii) identify candidate pathways, genes, and metabolites associated with different stages of the chilling response. This time-course analysis provides an integrated view of short-term chilling responses in tomato seedlings and a basis for subsequent functional studies.

## 2. Materials and Methods

### 2.1. Plant Materials and Chilling Treatment

The chilling-sensitive tomato cultivar ‘Moneymaker’ (accession LA2706) was obtained from the Tomato Genetics Resource Center (TGRC, University of California, Davis, CA, USA) and used in this study. Seeds were sown in 50-cell plug trays containing peat, vermiculite, and perlite (3:1:1, *v*/*v*/*v*) (Zunyi Liangu Agricultural Technology Co., Ltd., Zunyi, China). After germination, uniform seedlings were transplanted into plastic pots containing the same substrate and grown under controlled conditions of 25 ± 1 °C/18 ± 1 °C (day/night), approximately 70% relative humidity, and a 16 h light/8 h dark photoperiod. Cultures were incubated under light from 7:00 a.m to 23:00, followed by dark incubation thereafter, and a light intensity of approximately 50,000 lx.

At the four-leaf stage, uniform seedlings were transferred to a growth chamber (Model TP-R1000C, Zhejiang Top Cloud-Agri Technology Co., Ltd., Hangzhou, China) maintained at 4 °C. Leaf samples were collected at 0, 1, 3, 6, 12, and 24 h after the onset of chilling. Samples collected immediately before chilling began (0 h) served as the baseline. Each biological replicate comprised leaf tissues pooled from five independent plants; one leaf was collected from each plant, cut into small pieces, and thoroughly mixed to form one composite sample, with three such composite samples prepared per time point. The fourth to sixth leaves (counting from the bottom) were collected to ensure developmental consistency. Pots were randomly positioned within the growth chamber to minimize positional effects. Chilling treatment was started at 9:00 a.m. Each biological replicate comprised mixed leaf tissues from several individual plants, with destructive sampling applied during sample collection. Immediately after collection, leaf tissues were frozen in liquid nitrogen and stored at −80 °C until analysis. Separate samples were used for physiological measurements, RNA extraction and transcriptome sequencing, and metabolite extraction, followed by widely targeted metabolomic analysis. Representative phenotypic changes were photographed at each sampling time. The experiment was performed once with three independent biological replicates, and no fully independent repeat was conducted.

### 2.2. Determination of Antioxidant Enzyme Activities and MDA Content

Fresh leaf tissue (0.1 g) from each biological replicate was used for the physiological assays. Superoxide dismutase (SOD), peroxidase (POD), and catalase (CAT) activities and malondialdehyde (MDA) content were determined using commercial assay kits (Weiermin Biotechnology Co., Ltd., Suzhou, China) according to the manufacturer’s instructions. Three biological replicates were analyzed at each sampling time. SOD activity was expressed as U g^−1^ FW, POD activity as U min^−1^ g^−1^ FW, CAT activity as μmol min^−1^ g^−1^ FW, and MDA content as nmol g^−1^ FW.

### 2.3. RNA Sequencing and Transcriptomic Analysis

Leaf samples collected at 0, 1, 3, 6, 12, and 24 h were designated as CK, DW1, DW2, DW3, DW4, and DW5, respectively, with CK denoting the 0 h baseline. Three independent biological replicates were analyzed at each time point, yielding 18 samples for transcriptome sequencing. Total RNA was extracted using the Polysaccharides & Polyphenolics-rich Kit (Metware Biotechnology Co., Ltd., Wuhan, China). RNA concentration was measured using a Qubit 4.0 fluorometer (Thermo Fisher Scientific, Waltham, MA, USA), and RNA integrity was assessed using a Qsep400 Bioanalyzer (Bioptic Inc., New Taipei City, Taiwan, China); the RIN values ranged from 5.2 to 7.7 across all samples, with 15 of 18 samples exceeding 7.0 (Supplementary Table S3 and Figure S1). Library preparation was performed using the 3′-tagged RNA-seq method, which is tolerant to partially degraded RNA and is compatible with RIN values ≥ 5.0. Libraries were sequenced on an Illumina NovaSeq platform (Illumina Inc., San Diego, CA, USA) by Metware Biotechnology Co., Ltd. (Wuhan, China).

Raw reads were filtered using fastp (v0.23.2) to remove adapter sequences, reads containing ambiguous bases, and low-quality reads. The resulting clean reads were aligned to the tomato reference genome (*S. lycopersicum* TS3 genome assembly) using HISAT2 (v2.2.1) with default parameters, and gene expression was quantified using featureCounts (v2.0.3) based on the annotated gene models. Principal component analysis (PCA) and Spearman correlation analysis were used to assess overall transcriptomic variation and consistency among biological replicates. Differential gene expression at each chilling time point relative to the 0 h baseline was analyzed using the DESeq2 R package [13]. Genes with an absolute log_2_ fold change (|log_2_FC|) ≥ 1 and a false discovery rate (FDR) < 0.05 were classified as differentially expressed genes (DEGs). Gene Ontology (GO) analysis was used to characterize the biological functions represented by the DEGs [14], and Kyoto Encyclopedia of Genes and Genomes (KEGG) pathway enrichment analysis was used to identify significantly associated pathways [15].

### 2.4. Widely Targeted Metabolomic Analysis

Widely targeted metabolomic profiling was performed on leaf samples collected at 0, 1, 3, 6, 12, and 24 h. Three independent biological replicates were analyzed at each time point. Metabolite extraction and UPLC–MS/MS analysis were conducted by Metware Biotechnology Co., Ltd. (Wuhan, China) using a previously described widely targeted metabolomics workflow [16].

Briefly, freeze-dried leaf samples were ground to a powder, and 50 mg of each sample was extracted with 1.2 mL of −20 °C pre-cooled 70% aqueous methanol containing internal standards (250 μg/mL). Samples were vortexed for 30 s at 30 min intervals for six cycles, kept at 4 °C overnight, and then centrifuged at 12,000 rpm for 3 min. The supernatants were passed through a 0.22 μm membrane filter before UPLC–MS/MS analysis.

Chromatographic separation was performed on an ExionLC AD system (SCIEX, Framingham, MA, USA) with an Agilent SB-C18 column (1.8 μm, 2.1 mm × 100 mm; Agilent Technologies, Santa Clara, CA, USA). The mobile phase consisted of 0.1% formic acid in water (A) and 0.1% formic acid in acetonitrile (B), with a gradient of 5% B at 0 min, linearly increased to 95% B at 9 min, held for 1 min, then returned to 5% B within 1.1 min and equilibrated until 14 min. The flow rate was 0.35 mL/min, the column temperature was 40 °C, and the injection volume was 2 μL. Mass spectrometry was performed on an Applied Biosystems 4500 Q TRAP system (SCIEX, Framingham, MA, USA) equipped with an ESI Turbo Ion-Spray interface, operating in positive and negative ion modes with the following parameters: source temperature 500 °C; ion-spray voltage 5500 V (positive)/−4500 V (negative); ion source gas I, gas II, and curtain gas at 50, 60, and 25 psi, respectively; and high collision-activated dissociation. QQQ scans were acquired in MRM mode with nitrogen as the collision gas.

Metabolites were identified based on retention time and precursor/product ion pairs using the Metware in-house plant widely targeted metabolomics database (MWLB). Quantification was based on MRM peak areas. QC samples, prepared by mixing equal aliquots of all extracts, were injected every 10 samples. More than 85% of metabolites in QC samples had a CV < 0.5, and more than 75% had a CV < 0.3, indicating satisfactory analytical reproducibility. Metabolites with a QC CV ≥ 0.5 were excluded.

Principal component analysis (PCA) was used to evaluate global differences in metabolite profiles among samples. Orthogonal partial least-squares discriminant analysis (OPLS-DA) was used to derive variable importance in projection (VIP) scores. The OPLS-DA models were constructed for each chilling-treatment comparison versus the 0 h baseline and validated using 200 permutation tests, and the R^2^Y and Q^2^ values were used to evaluate model reliability. A Q^2^ > 0.5 was considered acceptable, and Q^2^ > 0.9 indicated excellent predictive ability.

Metabolites with VIP ≥ 1, |log_2_FC| ≥ 1 (i.e., fold change ≥ 2 or ≤0.5), and raw *p* < 0.05 were classified as differential accumulated metabolites (DAMs). FDR-adjusted *p* values (Benjamini–Hochberg method) were also calculated for all metabolites. Metabolites satisfying FDR < 0.05 are listed as a high-confidence DAM set in Supplementary Table S4.

We adopted a dual-reporting strategy for differential metabolite analysis. Although FDR correction was performed to account for multiple testing, applying FDR < 0.05 as an additional hard screening threshold would drastically reduce DAM counts for certain time-point comparisons; for instance, only two DAMs would remain at the 3 h time point, which prevents robust cross-time-point KEGG enrichment and time-series pathway-level comparison. Therefore, downstream pathway-level analyses (including Venn analysis, hierarchical clustering and KEGG enrichment) were performed using the primary DAM set filtered by VIP ≥ 1, |log_2_FC| ≥ 1, and raw *p* < 0.05, while the complete FDR-corrected results and FDR-filtered high-confidence DAM list are provided in Supplementary Table S4 for independent inspection and re-analysis by readers.

Each chilling time point was compared with the 0 h baseline. The identified DAMs were annotated against the KEGG database for subsequent pathway-level analysis [15].

### 2.5. Pathway-Level Comparison of Transcriptome and Metabolome Responses

The transcriptomic and metabolomic datasets were integrated at the pathway level. At each chilling time point, DEGs and DAMs identified relative to the 0 h baseline were mapped to KEGG pathways to identify pathways containing changes at both molecular levels. This comparison was based on shared pathway annotation rather than formal statistical integration. Temporal patterns of selected pathway-associated DEGs and DAMs were then examined to characterize temporal associations during chilling. Heatmaps were used to visualize relative gene-expression and metabolite-abundance patterns across the six sampling times. Because separate biological samples were used for transcriptomic and metabolomic analyses, gene–metabolite correlations were not calculated. The analysis was intended to identify candidate chilling-responsive pathways and molecular features, not to infer direct gene–metabolite regulation or metabolic flux.

### 2.6. Quantitative Real-Time PCR Validation

Eight representative DEGs showing different temporal expression patterns were selected for quantitative real-time PCR (qRT-PCR) analysis using a Roche LightCycler 96 Real-Time PCR System (Roche, Mannheim, Germany). Gene-specific primers were designed using NCBI Primer-BLAST (https://ncbi.nlm.nih.gov/tools/primer-blast/index.cgi, accessed on 20 September 2026) and synthesized by Sangon Biotech Co., Ltd. (Shanghai, China). Primer sequences and amplicon sizes are provided in Table 1. *SlACTIN* (*Solyc_TS3_03G078400*) was used as the reference gene [17].

Total RNA from the same biological samples used for RNA-seq was analyzed by qRT-PCR. First-strand cDNA was synthesized using the NovoScript Plus All-in-one First Strand cDNA Synthesis SuperMix (Novoprotein, Shanghai, China) according to the manufacturer’s instructions. qRT-PCR was performed on a Roche LightCycler^®^ 96 system using NovoStart^®^ SYBR qPCR SuperMix Plus (Novoprotein, Shanghai, China). Each 20-μL reaction contained 10 μL of 2× SYBR Green Master Mix, 0.4 μL of each primer (10 μM), 2 μL of cDNA, and 7.2 μL of nuclease-free water. Thermal cycling conditions were: 95 °C for 1 min, followed by 40 cycles of 95 °C for 10 s, 60 °C for 30 s, and 72 °C for 30 s, with a final melting curve analysis from 65 °C to 95 °C. Amplification efficiencies were calculated from standard curves, and melting curves showed a single peak for each primer pair. Three independent biological replicates were analyzed at each sampling time, with three technical replicates per biological replicate. Ct values from the technical replicates were averaged before relative expression was calculated using the 2^−ΔΔCt^ method, with the 0 h sample as the calibrator [18].

### 2.7. Statistical Analysis

Physiological and qRT-PCR data were obtained from three independent biological replicates and are reported as the mean ± standard deviation (SD). For qRT-PCR, technical replicates were averaged within each biological replicate before statistical testing. Differences among sampling times were assessed by one-way analysis of variance (ANOVA), followed by Tukey’s multiple-comparison test. *p* < 0.05 was considered statistically significant. Data were organized and analyzed using Microsoft Excel 2019 and SPSS Statistics 25.0, and figures were prepared in Origin 2024.

## 3. Results

### 3.1. Phenotypic Responses of Tomato Seedlings to Chilling Stress

The phenotypic responses of tomato seedlings to chilling are shown in Figure 1. Relative to the 0 h baseline (CK), seedlings exposed to 4 °C for 1 h (DW1) or 3 h (DW2) showed only slight leaf drooping, with no obvious loss of overall plant vigor. After 6 h of chilling (DW3), leaf wilting became more pronounced and was accompanied by visible chlorosis and reduced plant size. After 12 and 24 h (DW4 and DW5, respectively), seedlings showed severe leaf curling, extensive chlorosis, and marked morphological changes, and their growth appeared to be slowed.

Overall, visible chilling injury became progressively more severe with treatment duration, suggesting a time-dependent progression of chilling injury in tomato seedlings. These observations are qualitative; quantitative growth measurements (e.g., plant height, leaf area, biomass) were not performed in this study.

### 3.2. Dynamic Changes in Antioxidant Enzyme Activities and MDA Content

To characterize physiological changes during chilling exposure, the activities of SOD, POD, and CAT and the content of MDA were determined at the six sampling times (Figure 2).

SOD activity increased by 29.4% at 1 h relative to the 0 h control (632.71 vs. 488.81 U g^−1^ FW, *p* < 0.05). It then declined and remained at intermediate levels from 3 to 24 h. Tukey’s multiple-comparison test indicated that SOD activity at DW2–DW5 did not differ significantly from that at either CK or DW1.

POD activity followed a different temporal pattern. No significant differences were detected among CK, DW1, DW2, and DW3, for which POD activity remained between 19.14 and 23.87 U min^−1^ g^−1^ FW. POD activity then increased by approximately 2.0-fold at 12 h and 2.0-fold at 24 h relative to the 0 h control (43.40 and 43.80 U min^−1^ g^−1^ FW, respectively; *p* < 0.05).

CAT activity increased by 61.7% at 1 h and 42.5% at 3 h relative to the 0 h control (71.55 and 63.02 μmol min^−1^ g^−1^ FW, respectively; *p* < 0.05). It then declined by 30.8% and 40.3% at 6 and 12 h, respectively, both significantly lower than the CK value (30.60 and 26.41 μmol min^−1^ g^−1^ FW). At 24 h, CAT activity returned to a level comparable to the control (49.07 μmol min^−1^ g^−1^ FW, not significantly different from CK).

MDA content increased rapidly but transiently during early chilling. It increased by 33.8% at 1 h relative to the 0 h control (14.09 vs. 10.53 nmol g^−1^ FW, *p* < 0.05), then decreased by 10.9% and 11.9% at 3 and 6 h, respectively. MDA content subsequently increased slightly to 10.45 and 11.15 nmol g^−1^ FW at 12 and 24 h. Thus, MDA content did not increase progressively with chilling duration.

Notably, CAT activity at 6 and 12 h and MDA content at 6 h were lower than the 0 h baseline. These non-monotonic changes suggest a stage-dependent adjustment of the antioxidant system rather than a linear accumulation of oxidative damage. The early induction of SOD, CAT, and MDA indicates a rapid initial response, whereas the later increase in POD suggests a shift toward POD-mediated H_2_O_2_ scavenging. MDA content is also influenced by membrane repair and overall antioxidant capacity, so a transient decrease does not necessarily indicate the absence of oxidative stress.

Thus, the four physiological indicators followed distinct temporal trajectories over the 24 h treatment: SOD, CAT, and MDA changed rapidly during the early stage, whereas POD activity increased markedly at the later sampling times.

### 3.3. Transcriptomic Responses of Tomato Seedlings to Chilling

RNA sequencing was performed on 18 samples representing six sampling times, with three biological replicates per time point. Each library yielded more than 5.76 Gb of clean data, with Q30 values above 97.09% and GC contents ranging from 41.51% to 42.82%. More than 98.59% of clean reads mapped to the tomato reference genome, and uniquely mapped reads accounted for more than 93.5%. Spearman correlation coefficients among biological replicates exceeded 0.83, supporting the consistency of the transcriptomic dataset (Figure 3B).

PCA showed separation of samples by sampling time, whereas biological replicates from the same time point clustered relatively closely (Figure 3A). The first two principal components explained 52.13% of the total variation; PC1 and PC2 accounted for 33.94% and 18.19%, respectively. This pattern indicates substantial temporal variation in the transcriptome during the 24 h chilling treatment.

Differential expression analysis revealed extensive changes in gene expression relative to the 0 h baseline (Figure 3C). At 1 h, 4819 DEGs were identified, including 2286 upregulated and 2533 downregulated genes. The 3 h comparison yielded 4471 DEGs (2086 upregulated and 2385 downregulated), whereas the 6 h comparison yielded 4442 DEGs (2069 upregulated and 2373 downregulated). At 12 h, the DEG count increased to 5742, including 2906 upregulated and 2836 downregulated genes. The largest transcriptional response occurred at 24 h, when 8701 DEGs were detected, comprising 4358 upregulated and 4343 downregulated genes. Thus, DEG counts were similar during the first 6 h but increased substantially at 12 h and especially at 24 h, indicating expansion of the transcriptional response during later chilling.

Venn analysis identified 945 DEGs that were common to all five comparisons with the 0 h baseline (Figure 3D). This common DEG set represented genes responsive at every examined chilling time point and was used in subsequent functional analyses.

### 3.4. Functional Classification and Enrichment Analysis of Shared DEGs

To characterize the potential functions of the 945 DEGs shared across all five comparisons, GO functional classification and enrichment analyses were performed. The shared DEGs were assigned to 28 GO categories: 15 biological process (BP), two cellular component (CC), and 11 molecular function (MF) categories (Figure 4A). Major categories included cellular process (GO:0009987), metabolic process (GO:0008152), cellular anatomical entity (GO:0110165), binding (GO:0005488), and catalytic activity (GO:0003824). This classification describes the broad functional distribution of the shared DEGs and should not be interpreted as statistical enrichment.

GO enrichment analysis was then performed on the 945 shared DEGs, and the 20 most highly enriched terms are shown in Figure 4B. Enriched terms included responses to oxygen availability and hypoxia, ethylene-associated processes, and terpenoid metabolism. Oxidoreductase-related molecular functions were also represented. These findings associate the shared DEGs with multiple biological processes across the chilling time course.

KEGG analysis assigned the shared DEGs to 113 pathways, 14 of which were significantly enriched (Figure 4C). Prominent pathways included metabolic pathways (147 DEGs), biosynthesis of secondary metabolites (101 DEGs), carbon metabolism (48 DEGs), phenylpropanoid biosynthesis (18 DEGs), glyoxylate and dicarboxylate metabolism (10 DEGs), and plant–pathogen interaction (20 DEGs). Among these genes, 82 were annotated to both the ‘metabolic pathways’ and ‘biosynthesis of secondary metabolites’ categories and were selected for further analysis of their temporal expression patterns.

Hierarchical clustering of these 82 DEGs resolved two major expression patterns across the six sampling times (Figure 4D). One cluster showed relatively high expression in CK and DW1, followed by lower expression at subsequent sampling times. The second cluster showed relatively low expression in CK and DW1 but higher expression from DW2 to DW5, with several genes reaching particularly high relative expression at 24 h. These divergent patterns indicate that genes associated with major metabolic pathways responded differently over the course of chilling. The full list of DEGs at each time point is shown in Table S1.

### 3.5. Widely Targeted Metabolomic Responses During Chilling

Widely targeted metabolomic profiling was performed to characterize temporal changes in metabolite abundance during chilling. After filtering out metabolites with a QC CV ≥ 0.5, a total of 1150 metabolites were retained for downstream analysis. The CV distribution showed that more than 85% of metabolites in QC samples had a CV < 0.5 and more than 75% had a CV < 0.3 (Figure 5), indicating satisfactory analytical reproducibility.

Two-dimensional PCA showed that PC1 and PC2 explained 24.69% and 21.51% of the total variation, respectively, accounting for a cumulative 46.20% (Figure 6A). Biological replicates from the same sampling time generally clustered closely, whereas samples from different times separated in PCA space, indicating temporal variation in metabolite profiles during chilling.

OPLS-DA models were constructed for each chilling time point versus the 0 h baseline and validated using 200 permutation tests. The R^2^Y and Q^2^ values indicated reliable and predictive models (Supplementary Figure S3).

Differential accumulated metabolites (DAMs) for each comparison against the 0 h baseline were identified using the criteria of VIP ≥ 1, |log_2_FC| ≥ 1, and raw *p* < 0.05. Using this set of criteria, 262 DAMs were identified in DW1 versus CK, including 158 increased and 104 decreased metabolites (Figure 6B). The corresponding comparisons yielded 173 DAMs (108 increased and 65 decreased) in DW2 versus CK, 170 DAMs (135 increased and 35 decreased) in DW3 versus CK, 247 DAMs (190 increased and 57 decreased) in DW4 versus CK, and 195 DAMs (150 increased and 45 decreased) in DW5 versus CK. The largest number of DAMs was therefore detected at 1 h, and the total DAM count varied non-monotonically over the 24 h chilling period.

We applied Benjamini–Hochberg FDR correction for multiple testing across all detected metabolites. The high-confidence DAM subset satisfying FDR < 0.05 was extracted for each comparison: 33, 2, 95, 24, and 9 DAMs at 1, 3, 6, 12, and 24 h, respectively (Supplementary Table S4). Notably, only two DAMs passed the FDR < 0.05 threshold at the 3 h time point, which would preclude robust cross-time-point KEGG functional enrichment. We therefore adopted a dual-reporting strategy: the main-text analyses (including Venn analysis, clustering and KEGG enrichment) were performed based on DAMs filtered by VIP ≥ 1, |log_2_FC| ≥ 1 and raw *p* < 0.05, while the complete FDR-adjusted results and FDR-filtered high-confidence DAM list are fully provided in Supplementary Table S4 for independent inspection and re-analysis by readers.

Venn analysis identified 26 DAMs shared across all five comparisons; 23 increased and three decreased relative to the 0 h baseline (Figure 6C). These shared DAMs included six amino acid derivatives, six phenolic acids, five flavonoids, three lipids, two alkaloids, one lignan/coumarin, one organic acid, and two metabolites from other classes. Hierarchical clustering revealed distinct temporal abundance patterns among the 26 shared DAMs (Figure 6D). Several flavonoids, including kaempferol-3-O-sophoroside and cyanidin-3-O-(6″-O-acetyl)glucoside-5-O-glucoside, had relatively high abundance at 0 h and lower abundance during chilling. In contrast, pinoresinol-4-O-glucoside, cinnamoyltyramine, and 3-furoic acid were relatively more abundant at later sampling times.

Other shared metabolites exhibited transient temporal patterns. γ-Glu-Cys and cinnamoyltyramine decreased at 1 h and then increased from 3 to 24 h, whereas guanosine 3′,5′-cyclic monophosphate had relatively high abundance at 1 h followed by a gradual decrease. Several nucleoside-related metabolites were more abundant at the early sampling times, whereas some lipid-related metabolites increased at 12 and 24 h. The relative changes in DAMs across the five comparisons with the 0 h baseline are further visualized in Figure 6E.

KEGG pathway enrichment analysis of the DAMs identified several significantly enriched pathways, including metabolic pathways, biosynthesis of secondary metabolites, phenylpropanoid biosynthesis, tyrosine metabolism, biosynthesis of amino acids, glutathione metabolism, flavone and flavonol biosynthesis, and ABC transporters. Collectively, these results show that metabolite abundance changed rapidly during chilling and followed multiple, non-monotonic patterns rather than a uniform temporal progression. All identified DAMs are summarized in Table S2.

### 3.6. Pathway-Level Comparison of Transcriptomic and Metabolomic Responses

Direct comparison of the transcriptomic and metabolomic datasets revealed distinct temporal response patterns during chilling. The largest number of DAMs was detected at 1 h (262 DAMs), after which the number decreased and varied non-monotonically. In contrast, DEG counts remained similar from 1 to 6 h and then increased substantially, reaching 8701 at 24 h. These divergent profiles indicate partially asynchronous transcriptional and metabolic responses during the 24 h chilling treatment.

Pathway-level integration was performed by mapping DEGs and DAMs to KEGG pathways. Several pathways contained changes at both the transcript and metabolite levels, including metabolic pathways, biosynthesis of secondary metabolites, phenylpropanoid biosynthesis, tyrosine metabolism, biosynthesis of amino acids, glutathione metabolism, flavone and flavonol biosynthesis, and ABC transporters. Carbon metabolism, tyrosine metabolism, and phenylpropanoid biosynthesis were selected for detailed examination because they showed prominent chilling-responsive changes and linked primary and secondary metabolic processes.

Within carbon metabolism, *Solyc_TS3_11G002174*, annotated as encoding D-lactate dehydrogenase (D-LDH), showed decreased transcript abundance at 12 and 24 h relative to the 0 h baseline (Figure 7). In tyrosine metabolism, *Solyc_TS3_09G001641*, annotated as DOPA decarboxylase (DDC), was downregulated at 12 and 24 h, whereas *Solyc_TS3_05G000754*, annotated as a copper-containing amine oxidase (CuAO), showed downregulation at 3 h. These genes therefore exhibited distinct time-dependent transcriptional responses within the annotated metabolic pathways.

Several genes associated with phenylpropanoid biosynthesis also showed contrasting temporal patterns. The phenylalanine ammonia-lyase (PAL) gene *Solyc_TS3_05G002527* was downregulated at 1 h but showed increased expression from 3 to 24 h. The cinnamate 4-hydroxylase (C4H) gene *Solyc_TS3_05G001860* was downregulated at 3 h. In contrast, the cinnamoyl-CoA reductase (CCR) gene *Solyc_TS3_06G001372* showed an overall increase in expression with prolonged chilling exposure, whereas the cinnamyl alcohol dehydrogenase (CAD) gene *Solyc_TS3_02G001843* was downregulated from 6 to 24 h.

The integrated analysis therefore identified carbon metabolism, tyrosine metabolism, and phenylpropanoid biosynthesis as prominent candidate pathways associated with the time-dependent chilling response. The contrasting gene-expression patterns within these pathways, combined with temporal changes in metabolite abundance, provide candidate molecular features for further investigation. These pathway-level associations do not establish direct gene–metabolite regulation or changes in metabolic flux.

### 3.7. qRT-PCR Validation

To assess the expression patterns obtained by RNA-seq, eight representative DEGs with distinct temporal profiles were analyzed by qRT-PCR (Figure 8). The temporal trends obtained by qRT-PCR were significantly correlated with the RNA-seq data (Pearson *r* = 0.852, *p* < 0.01; Supplementary Figure S4), although fold-change magnitudes differed for some genes. This concordance supports the temporal expression patterns identified by RNA-seq.

## 4. Discussion

### 4.1. Temporal Adjustment of Physiological Responses During Chilling

Chilling can disrupt cellular redox homeostasis, and antioxidant enzymes are important components of plant responses to low-temperature stress [5]. In this study, SOD, CAT, and POD followed distinct temporal trajectories during the 24 h chilling treatment. SOD activity increased rapidly and reached its maximum at 1 h, whereas CAT activity was significantly higher than the 0 h baseline at 1 and 3 h. POD activity changed little during the first 6 h but increased markedly at 12 and 24 h. These patterns suggest that individual components of the antioxidant system may respond at different stages under the present chilling conditions, rather than following a uniform trajectory.

Previous studies indicate that jasmonate and melatonin signaling can modulate physiological responses to cold in tomato [19]. ABA-related signaling has also been implicated in antioxidant responses and cold tolerance [20], and sucrose metabolism has been linked to redox homeostasis during cold exposure [21]. In this context, the contrasting temporal patterns of SOD, CAT, and POD may reflect dynamic adjustment of antioxidant components during continued chilling. However, because ROS, H_2_O_2_, hormones, and soluble sugars were not measured directly, the enzyme-activity data do not provide direct evidence of changes in these processes.

MDA followed a distinct temporal pattern, increasing significantly at 1 h and then declining toward the baseline level. Because MDA is commonly used as an indicator of lipid peroxidation, this transient increase could indicate enhanced lipid peroxidation during the initial response to chilling. MDA and related membrane-injury traits have also been used to characterize cold responses in functional studies of tomato cold-regulatory genes [22]. Importantly, MDA did not increase progressively during prolonged chilling, suggesting that the physiological response observed over 24 h cannot be described as steadily increasing oxidative damage.

Tomato cold responses also involve diverse transcriptional and signaling components. Natural variation in the promoter of *SlWRKY33* has been associated with differences in cold sensitivity in tomato [23]. *SlWRKY50* has also been shown to enhance cold tolerance through jasmonic acid signaling [24]. In addition, salicylic acid-mediated protection of photosystem II contributes to the tomato response to chilling [25]. These studies emphasize the complexity of tomato cold responses and provide a broader context for the distinct temporal patterns observed here. Collectively, our physiological data point to a stage-dependent response characterized by rapid early changes in SOD, CAT, and MDA and a later increase in POD activity. However, because all time points were compared with the 0 h baseline without time-matched non-chilling controls, we cannot completely exclude the possibility that some of these temporal changes may be influenced by circadian or other time-dependent factors.

Notably, CAT activity at 6 and 12 h and MDA content at 6 h were lower than the 0 h baseline. These non-monotonic changes suggest a stage-dependent adjustment of the antioxidant system rather than a linear accumulation of oxidative damage. The later increase in POD may functionally compensate for the decline in CAT, and MDA content is also influenced by membrane repair and overall antioxidant capacity.

### 4.2. Transcriptomic and Metabolic Responses Display Partially Asynchronous Temporal Dynamics

The transcriptomic data revealed extensive, time-dependent reprogramming during chilling. Relative to the 0 h baseline, 4819, 4471, and 4442 DEGs were detected at 1, 3, and 6 h, respectively, showing that a substantial transcriptional response was established early in the treatment. DEG counts then increased to 5742 at 12 h and 8701 at 24 h. Thus, the pronounced increase at 12–24 h suggests further expansion of the transcriptional response during prolonged chilling.

The metabolomic response followed a different trajectory. The largest number of DAMs (262) was detected at 1 h, followed by 173, 170, 247, and 195 DAMs at 3, 6, 12, and 24 h, respectively. The metabolic response was therefore rapid but non-monotonic and did not mirror the later increase in DEG number. This divergence is biologically plausible because metabolite abundance depends not only on transcript abundance but also on pre-existing enzyme pools, substrate availability, transport, and post-transcriptional regulation.

Combined transcriptomic and metabolomic analyses in other plant species similarly demonstrate that cold responses involve extensive changes across multiple molecular layers. In kiwifruit, combined transcriptomic and metabolomic analysis identified coordinated changes in several metabolic pathways associated with freezing tolerance [26]. Combined analysis in common bean also revealed complex transcript–metabolite networks during cold stress [27]. Similar pathway-level coordination of transcriptional and metabolic responses has been reported in tobacco [28]. Comparative analysis of two potato cultivars further showed that cold-responsive transcriptomic and metabolic patterns can vary with genetic background [29].

Only 26 DAMs were shared across all five chilling comparisons, despite the large number detected at individual time points. These shared metabolites spanned several chemical classes rather than a single dominant group. When considered alongside the distinct temporal distributions of DEGs and DAMs, this diversity suggests partially asynchronous and heterogeneous molecular responses during short-term chilling. The present time-course data do not, however, establish a causal sequence from transcriptional regulation to metabolite accumulation. Accordingly, the transcriptomic and metabolomic datasets are more appropriately interpreted as convergent pathway-level changes than as direct one-to-one regulatory relationships between individual DEGs and DAMs. Furthermore, because the 0 h sample served as the sole baseline, the temporal patterns described here should be interpreted as changes associated with the chilling time course, which may also incorporate circadian or developmental influences.

### 4.3. Candidate Metabolic Pathways Associated with the Chilling Response

A pathway-level comparison of the transcriptomic and metabolomic datasets highlighted carbon metabolism, tyrosine metabolism, and phenylpropanoid biosynthesis as pathways containing both DEGs and DAMs. Because separate biological samples were used for the two analyses, this comparison should be regarded as an annotation-based overlap rather than a direct statistical integration. These pathways encompass both primary and specialized metabolism and may therefore reflect broad metabolic adjustment during low-temperature exposure. However, shared pathway annotation does not necessarily indicate direct regulatory relationships between individual DEGs and DAMs, and the absence of time-matched non-chilling controls means that some of the observed changes may also reflect circadian or developmental time-dependent effects.

Phenylpropanoid biosynthesis was particularly notable in the integrated analysis. The phenylpropanoid pathway produces diverse specialized metabolites and plays important roles in plant development and plant–environment interactions [30]. In the present study, PAL (*Solyc_TS3_05G002527*) was downregulated at 1 h but subsequently showed increased expression from 3 to 24 h, whereas C4H (*Solyc_TS3_05G001860*) was downregulated at 3 h. CCR (*Solyc_TS3_06G001372*) showed an overall increasing trend with prolonged chilling, while CAD (*Solyc_TS3_02G001843*) was downregulated from 6 to 24 h. These contrasting expression patterns indicate that individual components of phenylpropanoid biosynthesis responded differently over the chilling time course rather than undergoing uniform activation or repression.

The metabolomic dataset also contained both increased and decreased flavonoid- and phenolic acid-related metabolites. Heterogeneous changes in phenylpropanoid-derived compounds are consistent with the complex regulation of phenylpropanoid metabolism under abiotic stress [31]. Nevertheless, changes in the transcript abundance of PAL, C4H, CCR, or CAD alone cannot establish corresponding changes in enzyme activity, metabolic flux, lignin accumulation, or the production of specific downstream phenylpropanoid compounds. The present results therefore support the involvement of phenylpropanoid biosynthesis at the pathway-association level rather than demonstrating a defined regulatory mechanism.

Tyrosine metabolism also exhibited time-dependent transcriptional changes. The CuAO-associated gene *Solyc_TS3_05G000754* was downregulated at 3 h, whereas the DDC-associated gene *Solyc_TS3_09G001641* showed reduced expression at 12 and 24 h. When these gene-expression patterns are considered alongside the associated metabolite changes, tyrosine metabolism emerges as another candidate pathway in the temporal response to chilling. However, because CuAO and DDC activities and their specific metabolic products were not quantified independently, these findings do not demonstrate altered tyrosine-derived metabolic flux.

Carbon metabolism represents another pathway connecting the transcriptomic and metabolomic responses. *Solyc_TS3_11G002174*, annotated as a D-LDH-associated gene, was downregulated at 12 and 24 h, indicating a transcriptional response within this metabolic network during the later stage of chilling. However, D-LDH transcript abundance alone cannot be used to infer changes in carbon allocation or the supply of specific intermediates to downstream pathways. Therefore, conclusions regarding carbon flux into the shikimate or phenylpropanoid pathways would require additional biochemical evidence.

Overall, the integrated analysis identifies carbon metabolism, tyrosine metabolism, and phenylpropanoid biosynthesis as candidate pathways associated with short-term chilling responses in tomato seedlings. The temporal expression patterns of pathway-associated genes, considered alongside changes in related metabolite classes, define candidate molecular features for further functional investigation rather than a demonstrated regulatory network.

Because the present experiment used the 0 h samples as the baseline without time-matched non-chilling controls, some time-dependent changes may also reflect circadian or other temporal effects and therefore cannot be attributed exclusively to chilling. In addition, the experiment was performed once with three independent biological replicates, and no fully independent repeat was conducted. Furthermore, because separate biological samples were used for transcriptomic and metabolomic analyses, gene–metabolite correlations could not be directly assessed, and the pathway-level associations reported here should be interpreted with this limitation in mind. The experiment also examined a single tomato cultivar over a 24 h treatment period without a subsequent recovery or tolerance assessment. In addition, because only one chilling-sensitive cultivar was examined, the identified pathways cannot be considered determinants of chilling tolerance without comparison with a chilling-tolerant genotype or functional validation. Thus, the pathways, genes, and metabolites identified here should be interpreted as candidates associated with short-term chilling responses under the tested photoperiod.

## 5. Conclusions

Tomato seedlings exhibited stage-dependent physiological, transcriptional, and metabolic responses during 24 h of chilling at 4 °C. SOD and CAT activities and MDA content changed rapidly at early time points, whereas POD activity increased primarily at 12 and 24 h. The transcriptional response expanded markedly at later time points, reaching 8701 DEGs at 24 h; in contrast, the largest number of DAMs occurred at 1 h, indicating partially asynchronous transcriptional and metabolic dynamics. A pathway-level comparison highlighted carbon metabolism, tyrosine metabolism, and phenylpropanoid biosynthesis as prominent candidate pathways associated with chilling responses. Visible phenotypic changes, including leaf wilting, chlorosis, and curling, progressed with treatment duration; however, these observations are qualitative and quantitative growth measurements were not performed. These findings provide a time-resolved view of short-term chilling responses in tomato seedlings and identify genes, metabolites, and pathways for subsequent functional validation, although these candidates should be considered associated with chilling responses rather than determinants of chilling tolerance given the use of a single chilling-sensitive cultivar.

## Figures and Tables

**Figure 1:**
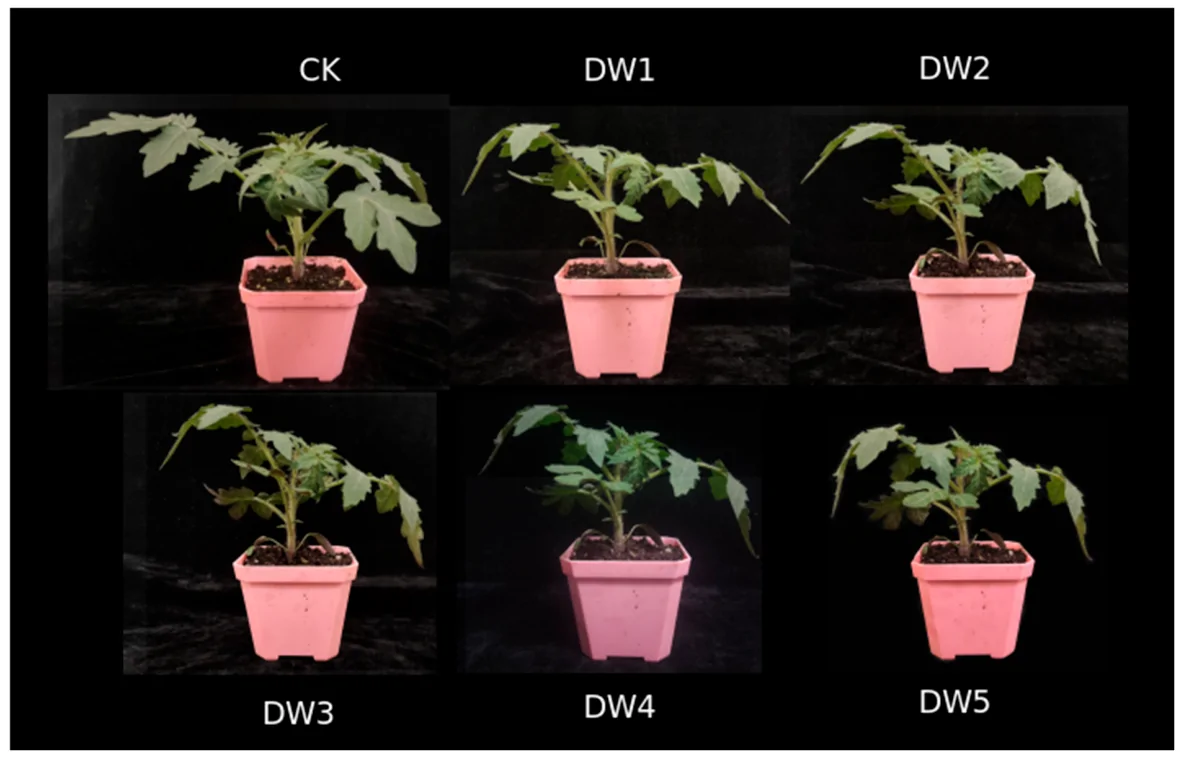
Phenotypic alterations of tomato seedlings under chilling stress at different treatment durations. CK, 0 h (before chilling); DW1, 1 h; DW2, 3 h; DW3, 6 h; DW4, 12 h; and DW5, 24 h after the onset of chilling. Photographs were taken with a black cloth as the original background. Only brightness was adjusted uniformly across all panels to compensate for slight differences in lighting during photography; no background replacement, cropping of plant material, or other content modification was performed. Each panel was cropped to the same aspect ratio and assembled in a 2 × 3 layout.

**Figure 2:**
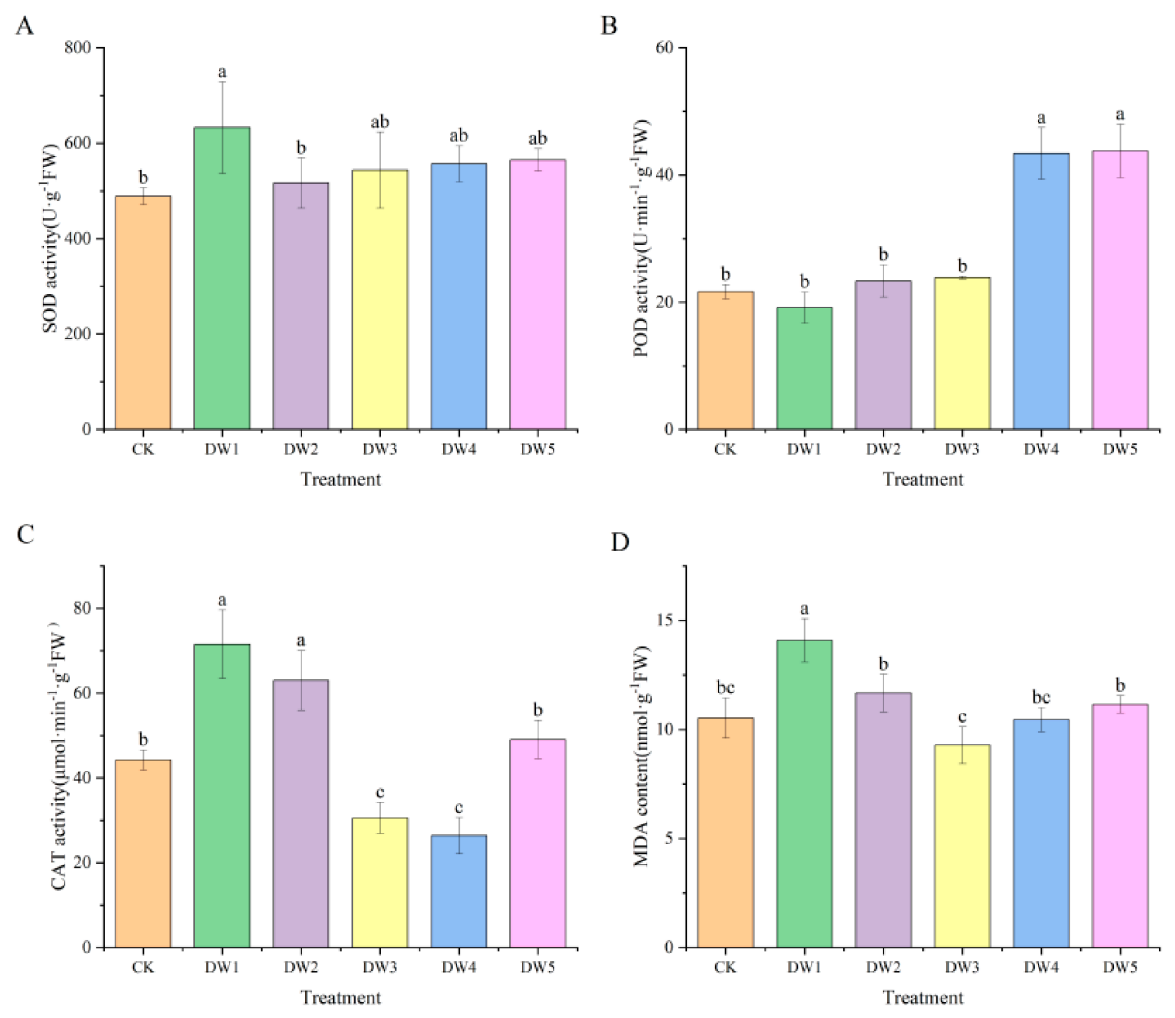
Changes in antioxidant enzyme activities and MDA content during chilling treatment. (**A**) SOD activity; (**B**) POD activity; (**C**) CAT activity; and (**D**) MDA content. Values are means ± SD (*n* = 3 independent biological replicates). Different lowercase letters indicate significant differences among sampling times based on Tukey’s multiple-comparison test (*p* < 0.05). The original values (mean ± SD) and significance letters for Figure 2 are provided in Supplementary Table S6.

**Figure 3:**
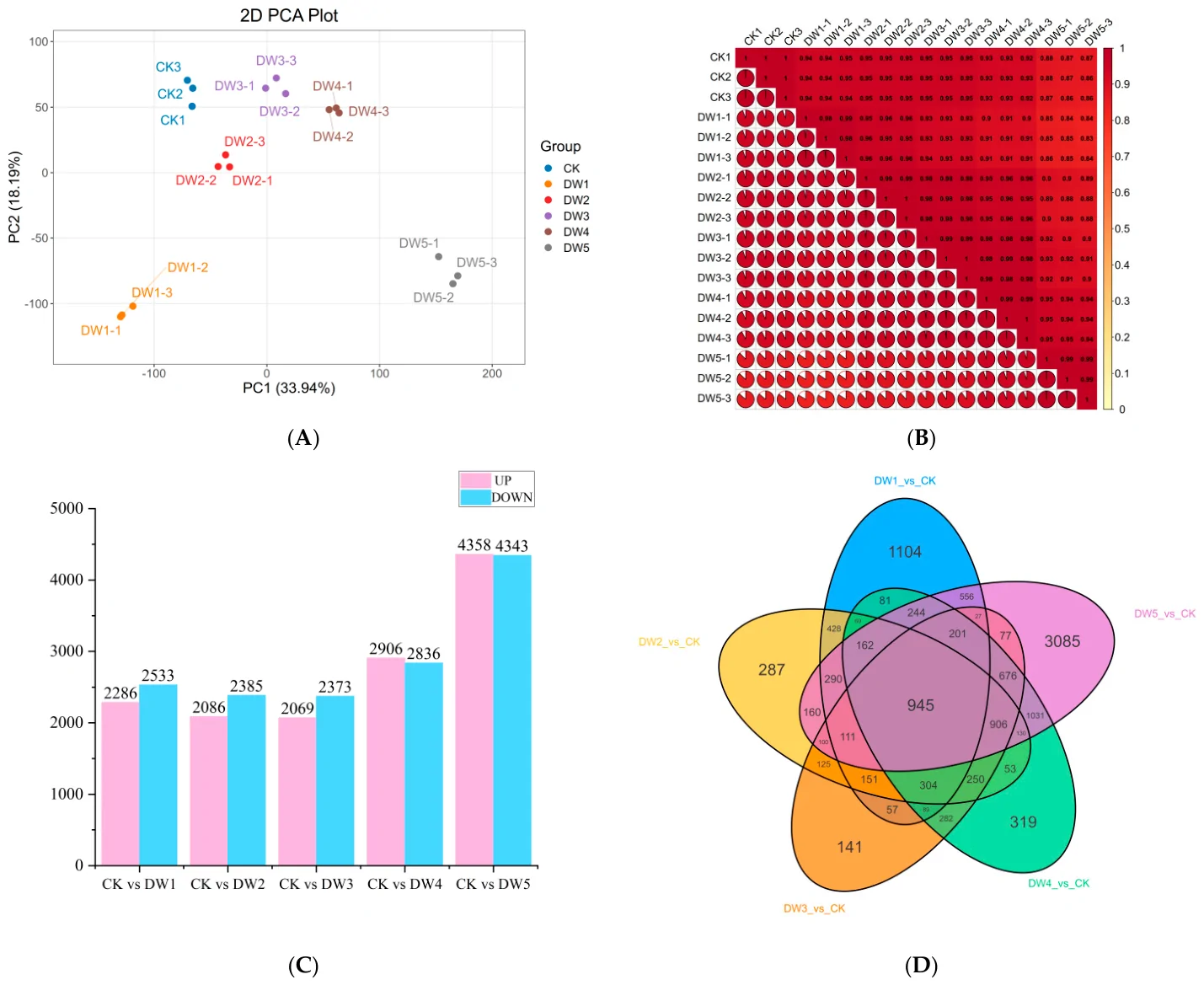
Global transcriptomic responses of tomato seedlings during chilling treatment. (**A**) Principal component analysis (PCA) of the 18 RNA-seq samples based on FPKM values, with data UV-scaled before analysis; (**B**) Spearman correlation matrix among samples calculated from FPKM values; (**C**) numbers of upregulated and downregulated DEGs at each chilling time point relative to the 0 h baseline using DESeq2 with |log_2_FC| ≥ 1 and padj < 0.05; and (**D**) Venn diagram showing DEGs shared among the five comparisons. Each time point includes three independent biological replicates (*n* = 3).

**Figure 4:**
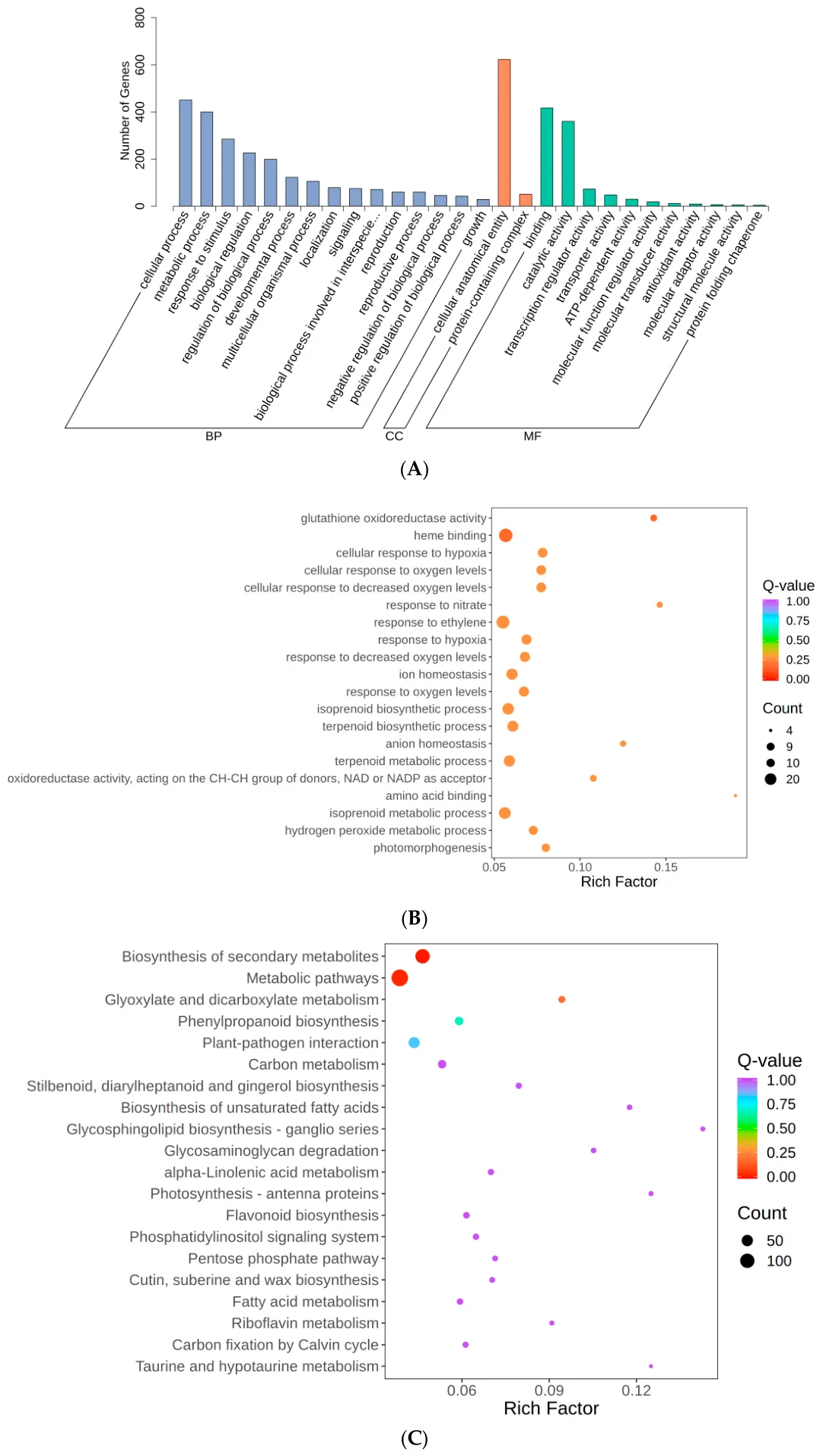

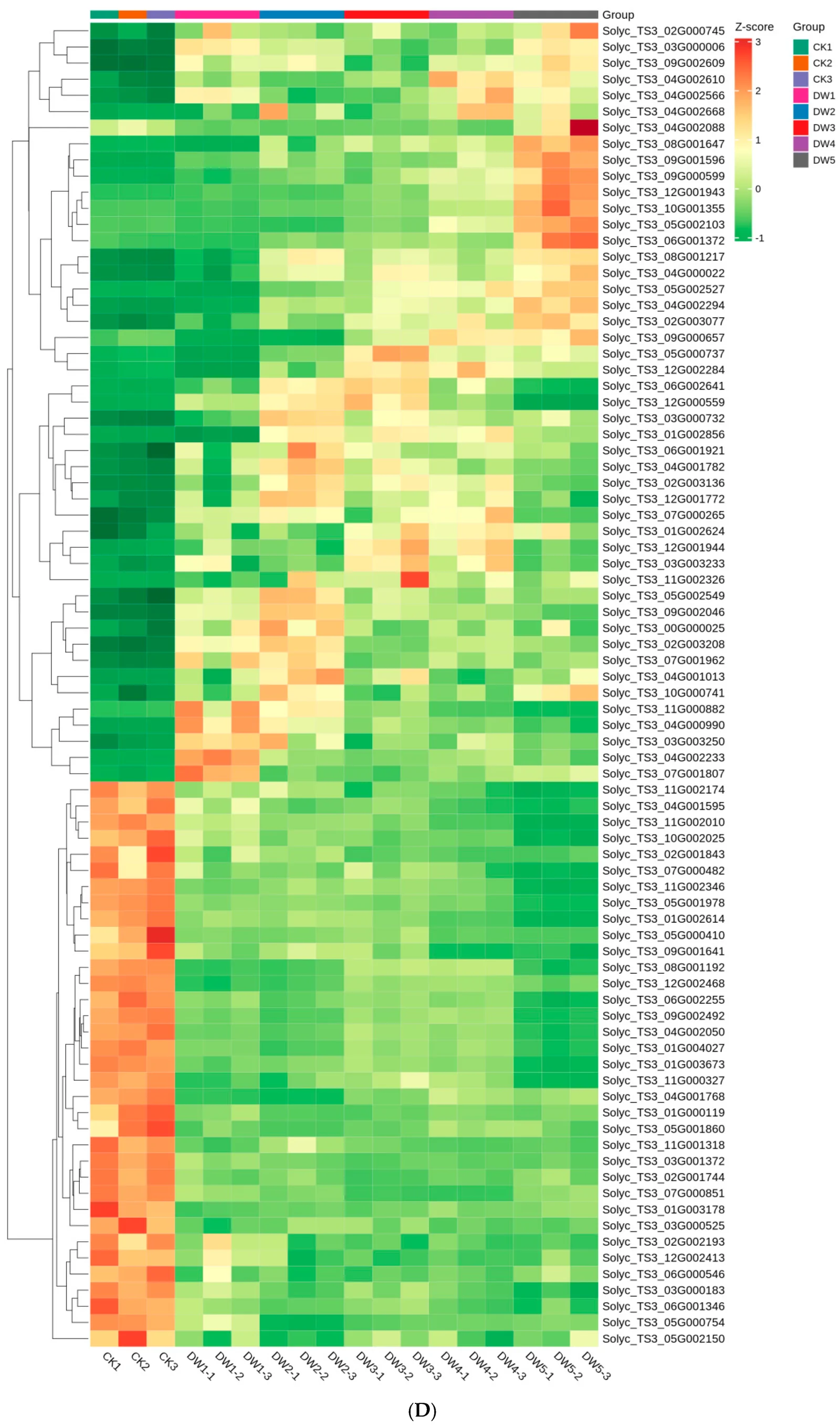
Functional classification and enrichment analysis of shared DEGs during chilling treatment. (**A**) GO functional classification of the 945 shared DEGs; (**B**) GO enrichment analysis of the shared DEGs using the hypergeometric test, with *p* values adjusted by the hypergeometric test, with *p* values adjusted by the Benjamini–Hochberg method (padj < 0.05); (**C**) KEGG pathway enrichment analysis of the shared DEGs using the hypergeometric test, with *p* values adjusted by the Benjamini–Hochberg method (padj < 0.05); and (**D**) heatmap showing the temporal expression patterns of 82 DEGs associated with metabolic pathways and biosynthesis of secondary metabolites, based on Z-score-normalized FPKM values, with hierarchical clustering using complete linkage and Euclidean distance. Each time point includes three independent biological replicates (*n* = 3).

**Figure 5:**
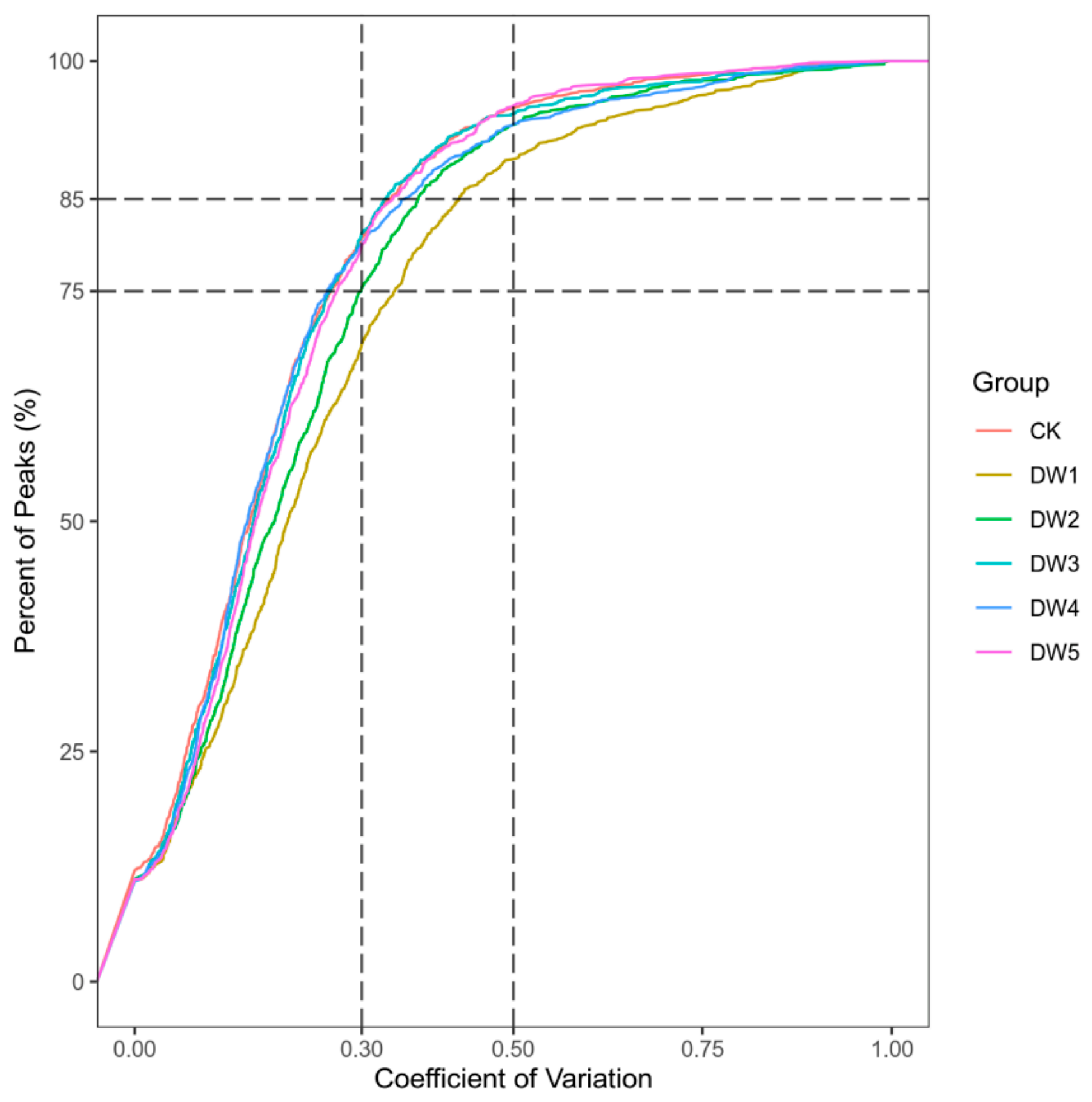
Coefficient of variation (CV) distribution of metabolites in QC and experimental samples. Empirical cumulative distribution function (ECDF) of the coefficient of variation (CV) for metabolite intensities across QC and experimental groups. The x-axis represents the CV value, and the y-axis represents the percentage of metabolites with a CV below the corresponding value. Dashed vertical lines indicate CV = 0.3 and CV = 0.5; dashed horizontal lines indicate 75% and 85%. For QC samples, more than 85% of metabolites had a CV < 0.5, and more than 75% had a CV < 0.3, indicating satisfactory analytical reproducibility and data stability. Each group contains three biological replicates (*n* = 3), except for the QC sample, which was prepared by pooling aliquots of all samples.

**Figure 6:**
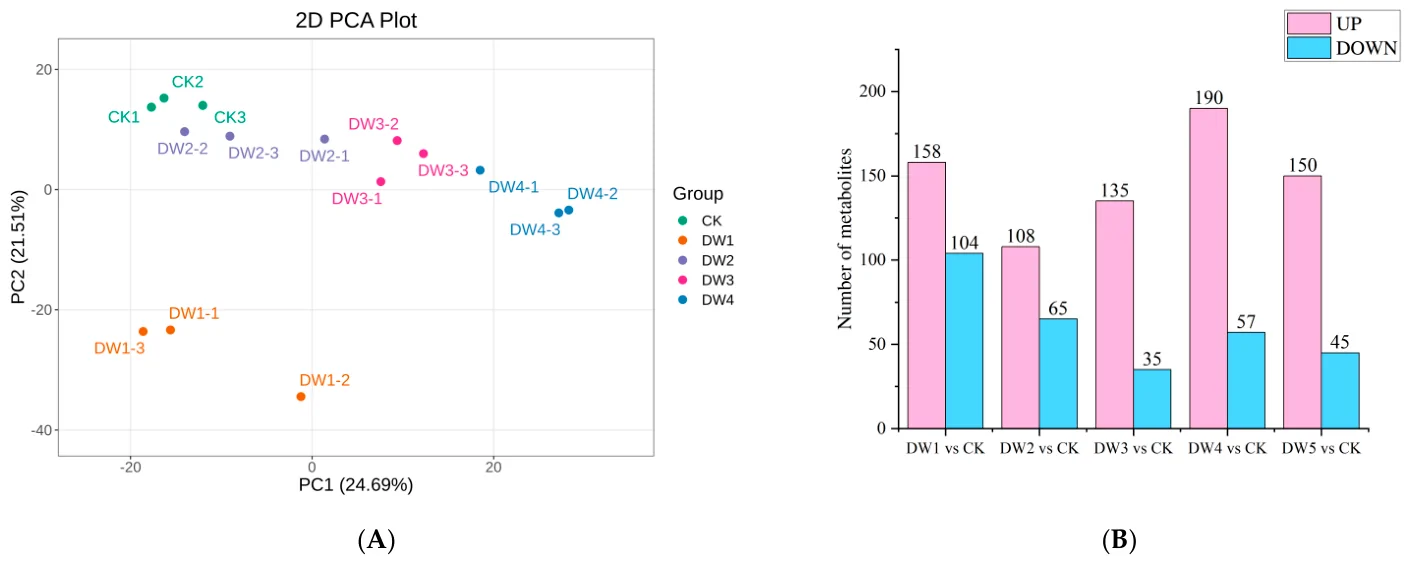

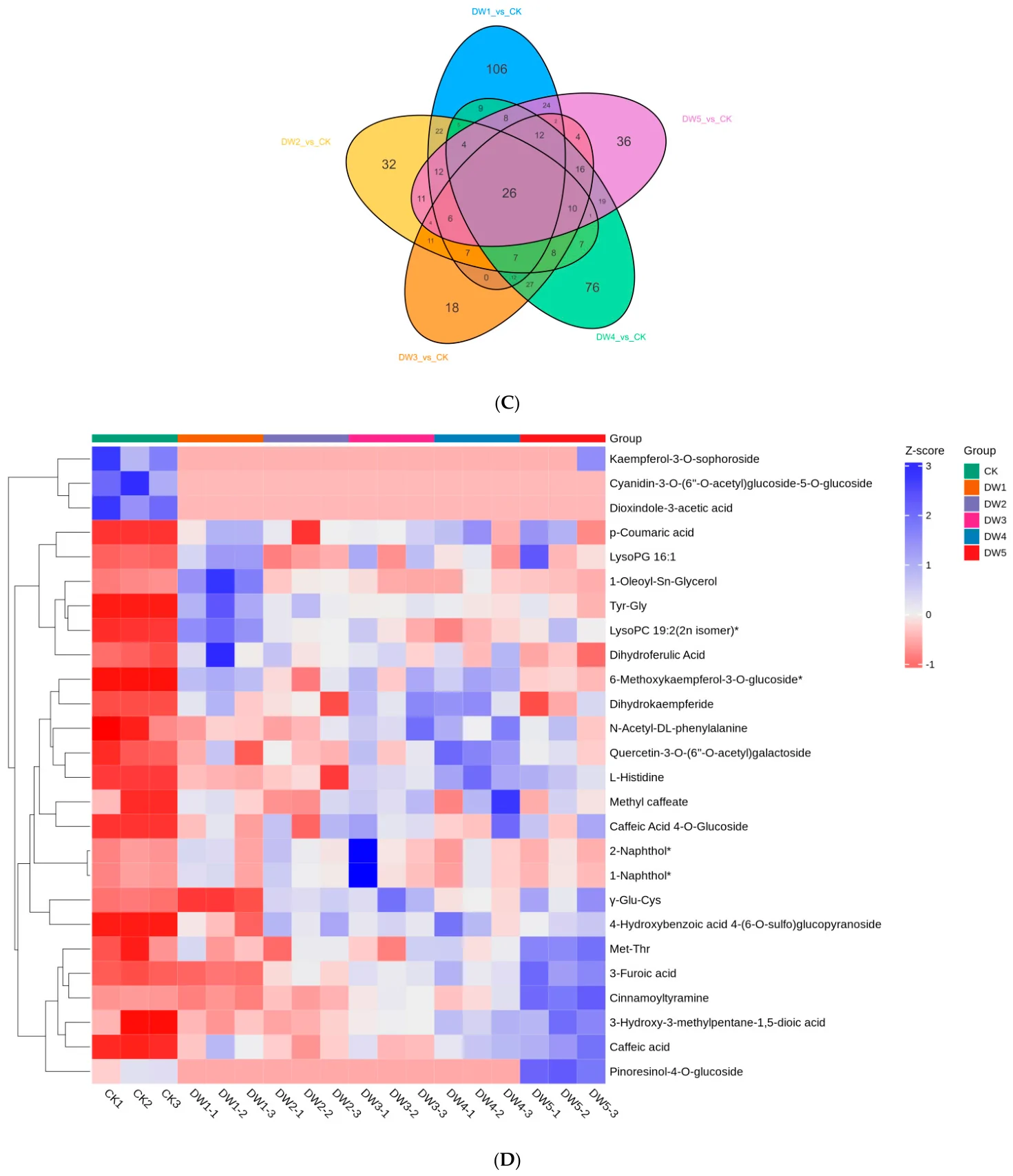

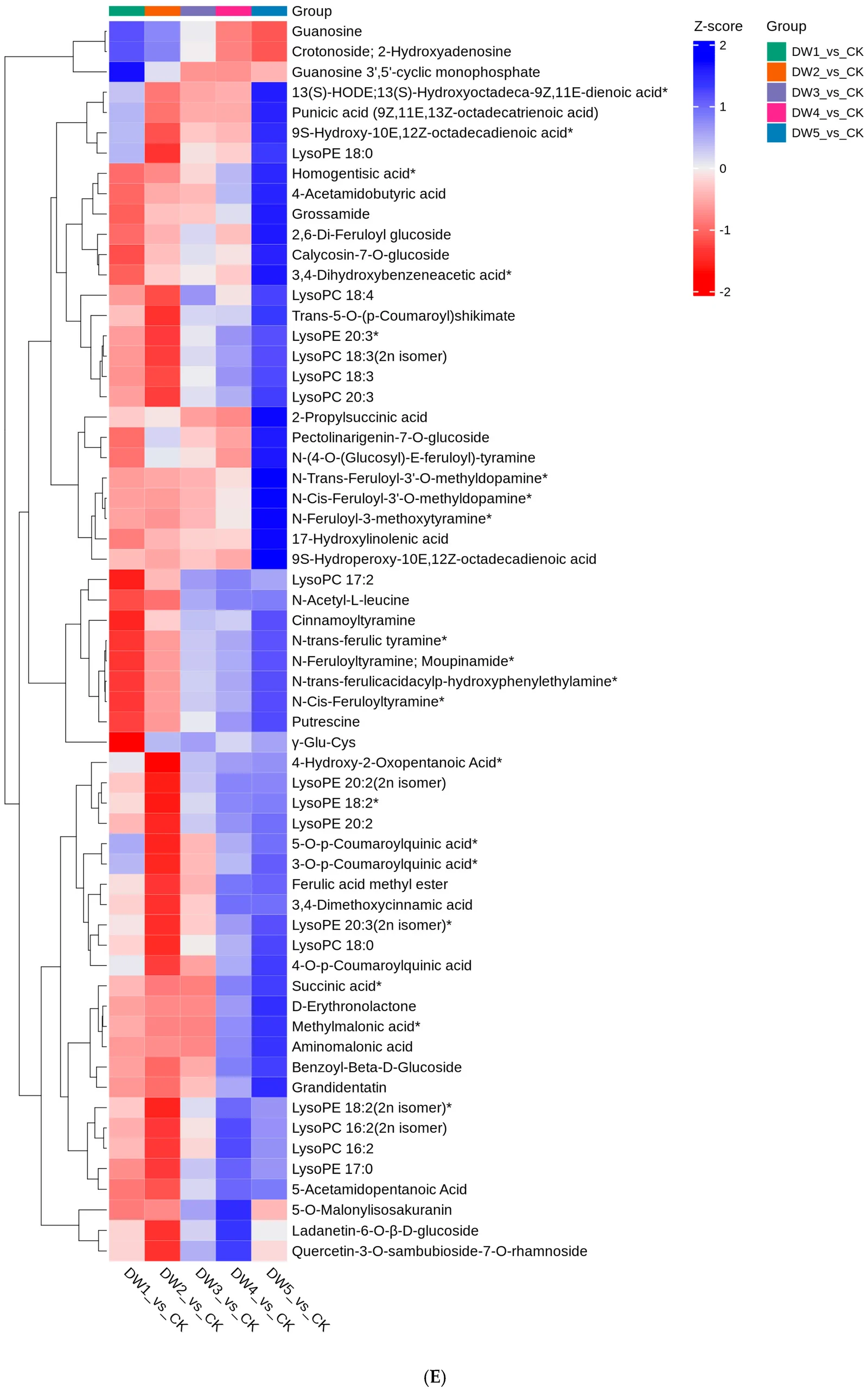
Widely targeted metabolomic profiling of tomato seedlings during chilling treatment. (**A**) Two-dimensional PCA score plot of metabolomic samples based on UV-scaled data; (**B**) numbers of increased and decreased DAMs at each chilling time point relative to the 0 h baseline, identified using VIP > 1 and |log_2_FC| ≥ 1; (**C**) Venn diagram showing shared and time-specific DAMs among the five comparisons; (**D**) hierarchical clustering heatmap of the 26 shared DAMs based on UV-scaled data, with hierarchical clustering using complete linkage and Euclidean distance; and (**E**) heatmap showing the relative changes in DAMs across the five comparisons with the 0 h baseline based on UV-scaled data. Each time point includes three independent biological replicates (*n* = 3).Metabolites marked with an asterisk (*) were putatively annotated without authentic reference standards.

**Figure 7:**
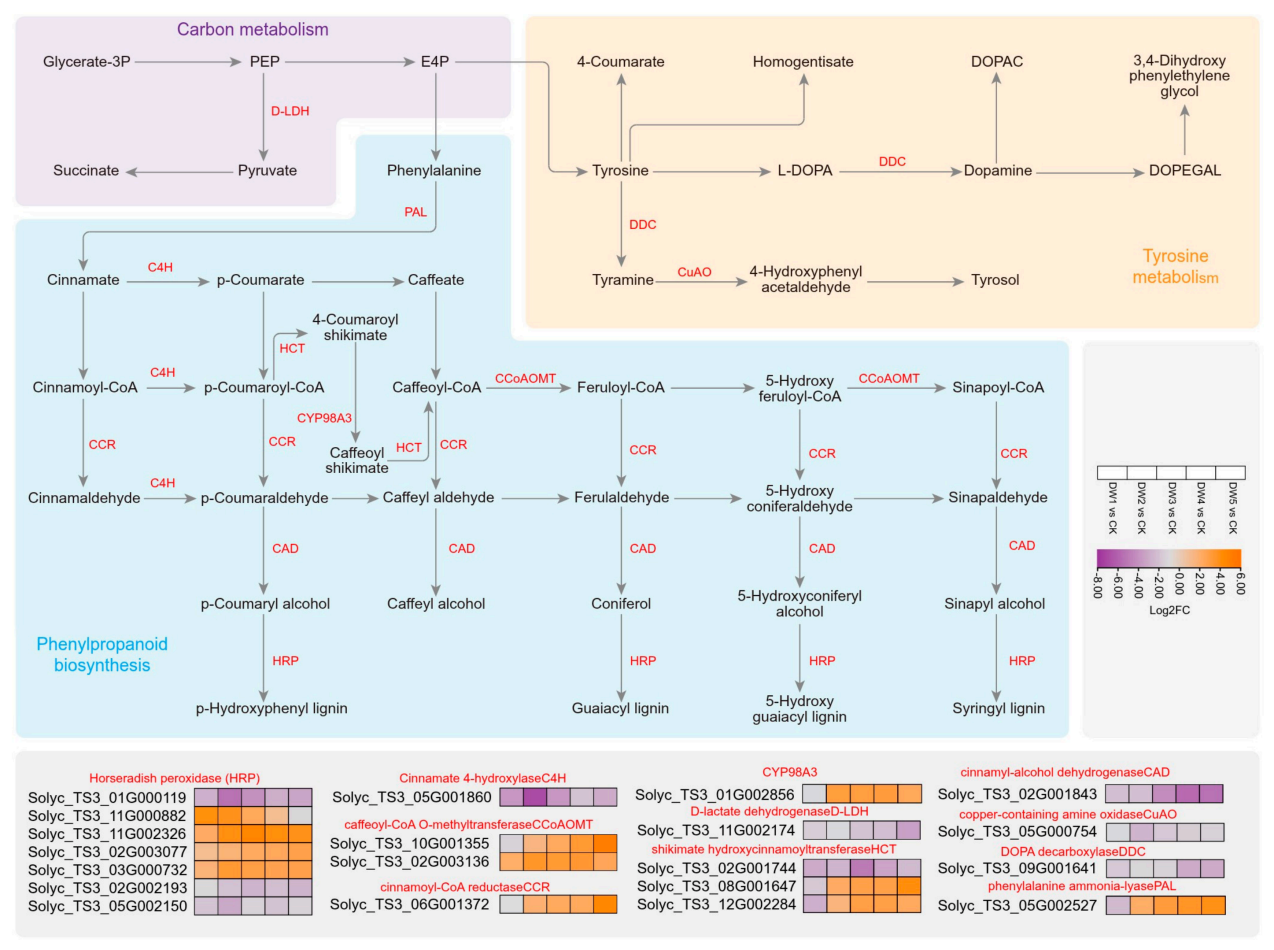
Pathway-level integration of selected chilling-responsive genes associated with carbon metabolism, tyrosine metabolism, and phenylpropanoid biosynthesis. Heatmaps show log_2_(fold change) values of selected genes at 1, 3, 6, 12, and 24 h relative to the 0 h baseline. DEGs were identified using DESeq2 with |log_2_FC| ≥ 1 and padj < 0.05. Pathway associations are based on functional annotation and do not imply direct regulatory relationships or metabolic flux. Each time point includes three independent biological replicates (*n* = 3).Arrows indicate the direction of biochemical reactions in the metabolic pathway.

**Figure 8:**
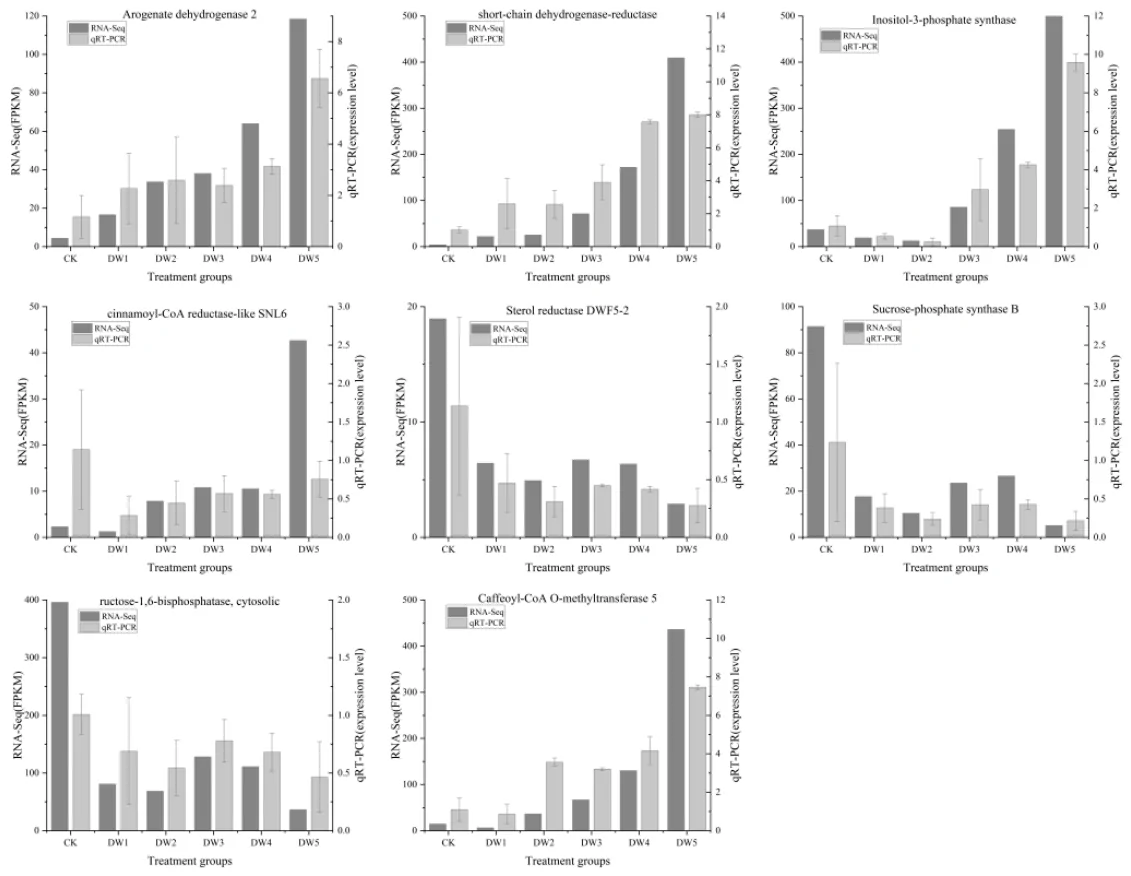
Comparison of the temporal expression patterns of eight selected DEGs measured by RNA-seq and qRT-PCR during chilling. RNA-seq data are presented as log_2_(fold change) values relative to the 0 h baseline. Relative qRT-PCR expression was calculated using the 2^−ΔΔCt^ method with SlACTIN as the reference gene and the 0 h sample as the calibrator. Values are means ± SD (*n* = 3 independent biological replicates, with three technical replicates each). The correlation between RNA-seq and qRT-PCR log_2_FC values was assessed using Pearson correlation analysis (*r* = 0.852, *p* < 0.01).

**Table 1:** Primers used for qRT-PCR analysis.

Gene ID	Gene Name	Full Gene Name	Primer Sequences (5′–3′)
*Solyc_TS3_03G078400*	*SlACTIN*	SlACTIN	F:GGACTTGCAACCCAAAGCTGT R:CCCATCAGGAAGCTCGTAGCT
*Solyc_TS3_09G000599*	*LOC101259361*	Arogenate dehydrogenase 2	F:CCCTCTCCGTGCCCAAAATA R:TGGATGGGAAGGGAACGAATG
*Solyc_TS3_12G001943*	*LOC100750250*	Short-chain dehydrogenase-reductase	F:GTAACGCTGGTGTAGCTGGT R:GCCGCACTTGTCGTGAAAAT
*Solyc_TS3_10G001355*	*LOC101253032*	Caffeoyl-CoA O-methyltransferase 5	F:ATGGAATGGGTCTGTGGCTG R:GATCAGATGATACGGCGGCA
*Solyc_TS3_05G002103*	*LOC543809*	Inositol-3-phosphate synthase	F:CCATGTGCCAAAATTGGGGG R:GGGTTGACCATGGGAAGGAG
*Solyc_TS3_06G001372*	*LOC101265652*	cinnamoyl-CoA reductase-like SNL6	F:CCAGCCTCAGATGATGGACC R:TGGAATTGGGGGTTTGGGAA
*Solyc_TS3_06G002255*	*DWF5-2*	Sterol reductase DWF5-2	F:CCACTGCAATTGCGGGAAAA R:GAAGGAGCCTTTCCCCAGAC
*Solyc_TS3_09G002492*	*LOC101055573*	Sucrose–phosphate synthase B	F:ACAGGTCGCAAGAGTGTCAG R:CGTGTCTCCAGTTTCACCGA
*Solyc_TS3_04G002050*	*LOC101266629*	ructose-1,6-bisphosphatase, cytosolic	F:TGCCAAGAACTGGGATGGTC R:CTGCACCAGCAAGAGTAGTGA

## Data Availability

The raw RNA-seq sequencing data generated in this study have been submitted to the NCBI Sequence Read Archive (SRA). The corresponding BioProject accession number will be added to the manuscript as soon as it is assigned by NCBI. The metabolomic raw data, metabolite annotation tables, processed gene-expression matrix, and differential analysis results are available within the article and its Supplementary Information Files. All other relevant raw data are available from the corresponding author upon reasonable request.

## Supplementary Materials

The following supporting information can be downloaded at: https://www.mdpi.com/article/10.3390/biom16101390/s1, Table S1: Complete list of differentially expressed genes (DEGs) across all chilling-treatment time points; Table S2: Complete list of differentially abundant metabolites (DAMs) across all chilling-treatment time point; Table S3: RNA sample quality metrics, including RNA integrity number (RIN) values of the samples used for transcriptome sequencing; Table S4: Complete list of differentially accumulated metabolites (DAMs) across all chilling-treatment groups; Table S5: Complete list of differentially expressed genes (DEGs) across all chilling-treatment groups; Table S6: Original physiological data (antioxidant enzyme activities and MDA content) used to generate Figure 2; Figure S1: Electropherograms of RNA samples used for transcriptome sequencing. Representative electropherograms showing 18S and 28S rRNA peaks for 18 RNA samples. The 28S/18S ratios and RIN values confirm high RNA integrity; all samples satisfied RIN ≥ 7.0 for library construction; Figure S2: Quality-control assessment of the UPLC–MS/MS system. (A) Extracted-ion chromatograms (XICs) of metabolite mws1090 (positive-ion mode); (B) XICs of metabolite pmf0440 (negative-ion mode); (C) Overlaid total-ion chromatograms (TICs of QC samples, positive-ion mode); (D) Overlaid TICs of QC samples, negative-ion mode. Consistent peak shapes, retention times and high overlap of QC chromatograms demonstrate good analytical reproducibility; Figure S3: OPLS-DA permutation tests for five comparisons (DW1–DW5 vs. CK). Histograms show the distribution of R^2^Y and Q^2^ values from 200 permutations. Original models exhibited Q^2^ = 0.902–0.947 (*p* < 0.005), indicating no model over-fitting; Figure S4: Correlation between RNA-seq and qRT-PCR results. Scatter plot of log_2_FC values from RNA-seq (x-axis) versus qRT-PCR (y-axis) for eight genes across all chilling time points (n = 48). Linear regression, Pearson correlation (r = 0.852) and *p*-value (*p* < 0.01) are presented; Figure S5: Unmodified original photographs of tomato seedlings for Figure 1. Seedling images taken at 0 h (CK), 1 h (DW1), 3 h (DW2), 6 h (DW3), 12 h (DW4), and 24 h (DW5) under 4 °C chilling. No cropping, brightness adjustment or background editing was performed on these raw photographs, provided for verification of Figure 1.
