# Supplementary material for: Time-Resolved Transcriptomic and Widely Targeted Metabolomic Profiling of Tomato Seedlings During Short-Term Chilling
Source: Biomolecules. 2026 Sep 23;16(10):1390. doi: 10.3390/biom16101390 (PMC13702160; doi:10.3390/biom16101390)
Supplement: Supplementary file 1 [file biomolecules-16-01390-s001.zip › Figures S1-S5.pdf]

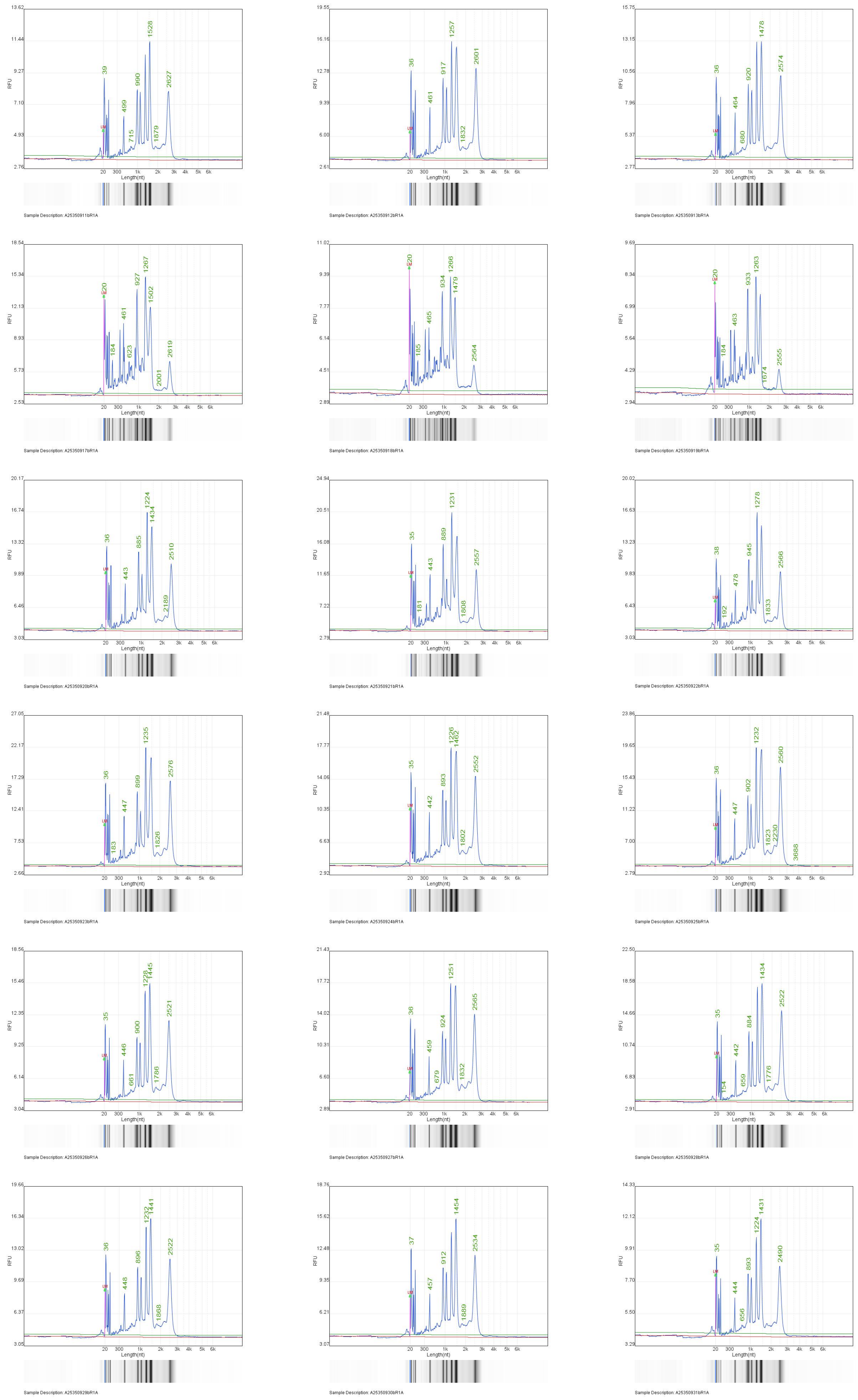

Supplementary Figure S1. Electropherograms of RNA samples used for

transcriptome sequencing. Representative electropherograms showing the 18S and 28S ribosomal RNA peaks for the 18 RNA samples. The 28S/18S ratios and RIN values indicate high RNA integrity. All samples met the RIN ⩾ 7.0 criterion for library preparation.

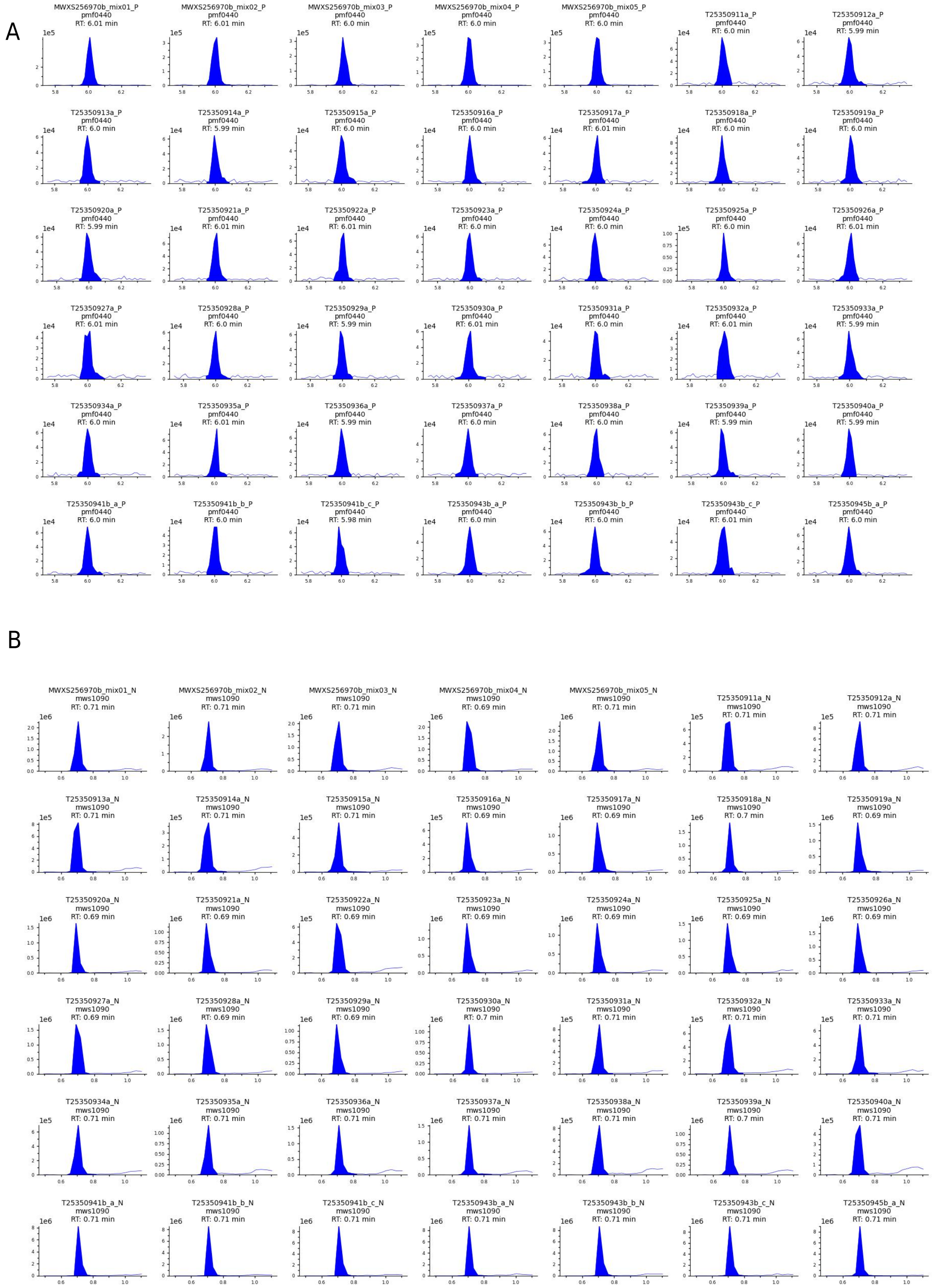

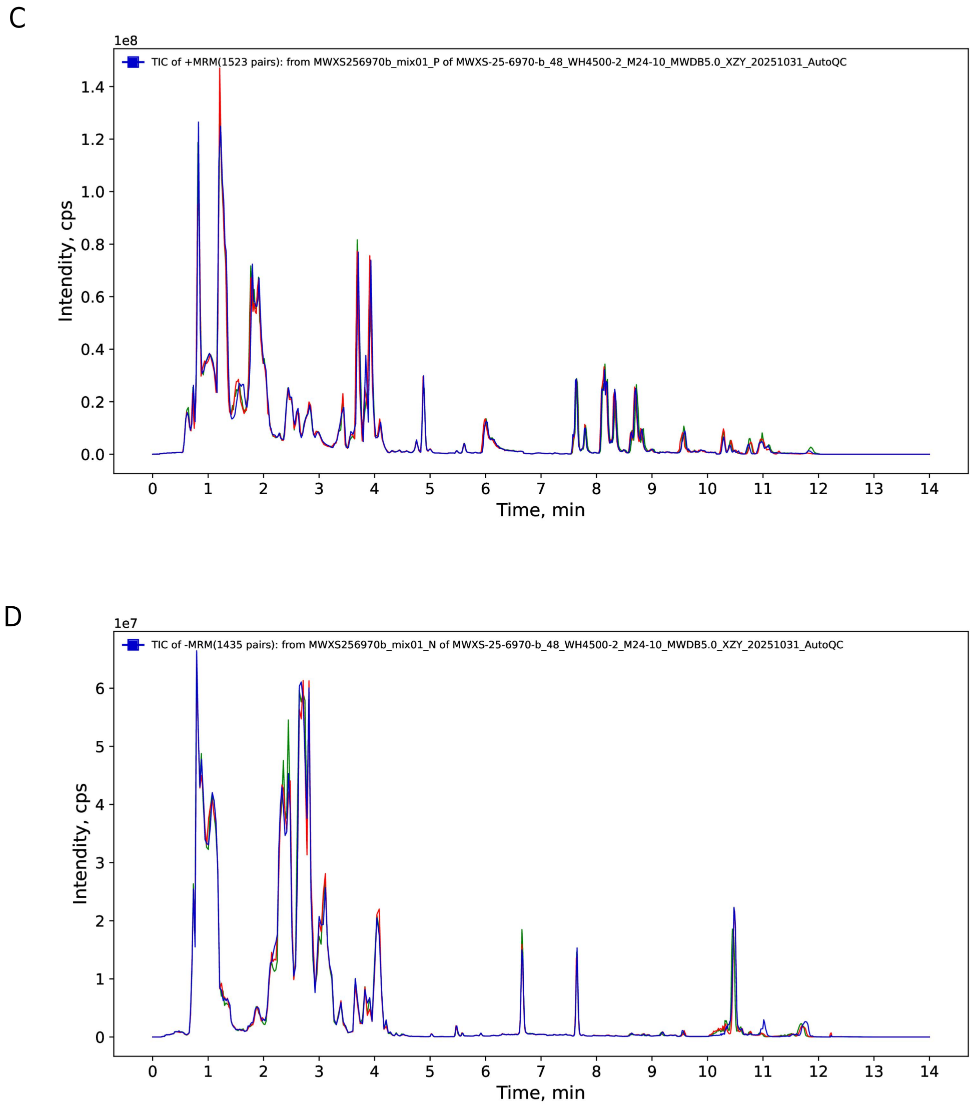

Supplementary Figure S2. Quality control assessment of the UPLC–MS/MS system.

(A) Extracted ion chromatograms (XICs) of a representative metabolite (mws1090) in positive ion mode across all QC and experimental samples. (B) XICs of a representative metabolite (pmf0440) in negative ion mode across all QC and experimental samples. (C) Overlaid total ion chromatograms (TICs) of QC samples in positive ion mode (−MRM, 1,435 ion pairs). (D) Overlaid TICs of QC samples in negative ion mode (+MRM, 1,523 ion pairs). For (A) and (B), each panel represents one sample, with the metabolite ID, ion mode, and retention time (RT) indicated. The consistent peak shapes and retention times across samples indicate satisfactory chromatographic stability. For (C) and (D), multiple QC injections were overlaid; the high degree of overlap in retention time and peak intensity indicates good reproducibility of the analytical system.

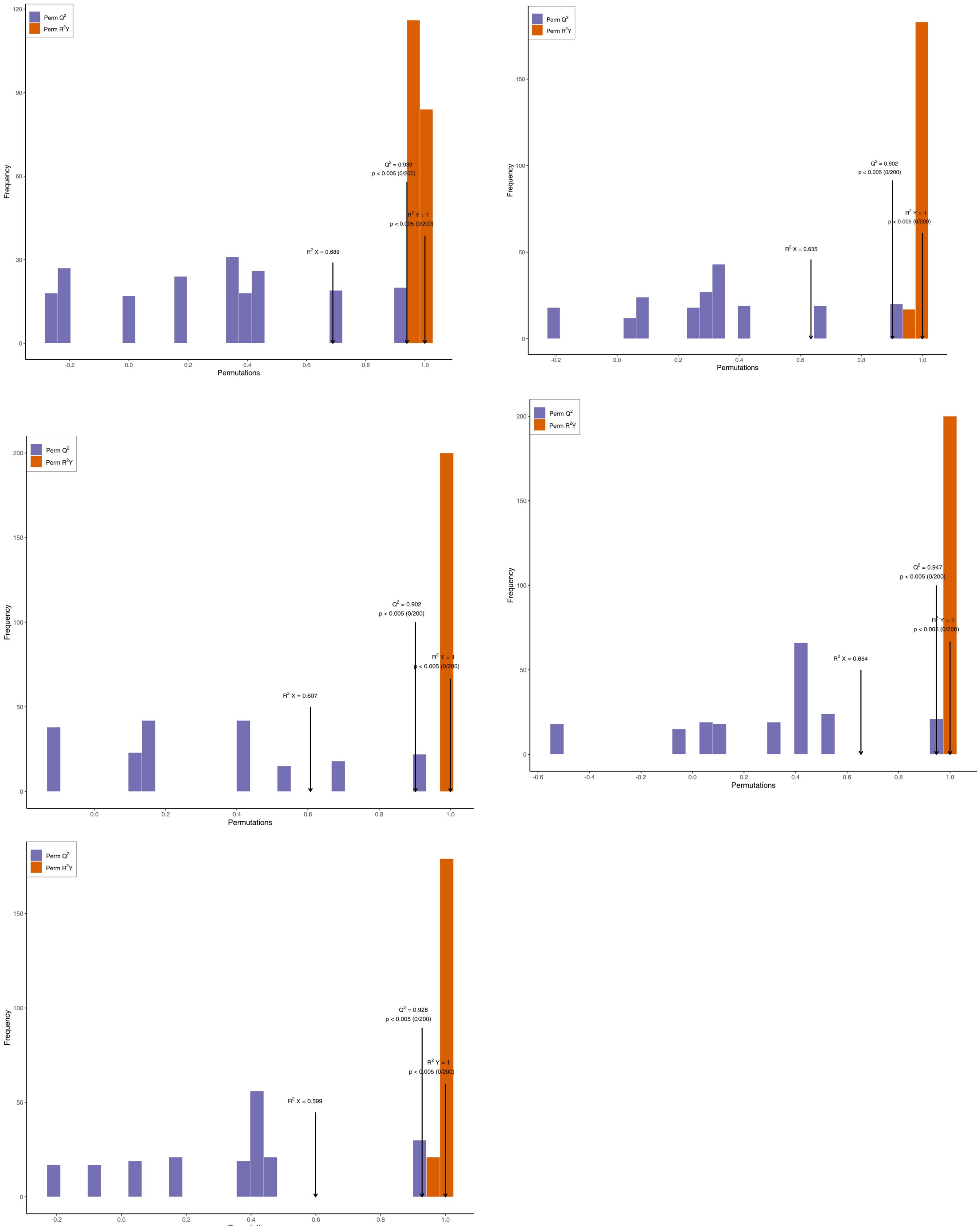

Supplementary Figure S3. OPLS-DA permutation tests for the five comparisons (DW1 – DW5 vs. CK). Histograms show the distribution of R^2Y and Q^2 values from 200 permutation tests. The original models (indicated by arrows) showed significantly higher Q^2 values (0.902 – 0.947) than all permuted models ($p < 0.005$, 0/200), demonstrating that the models were not overfitted.

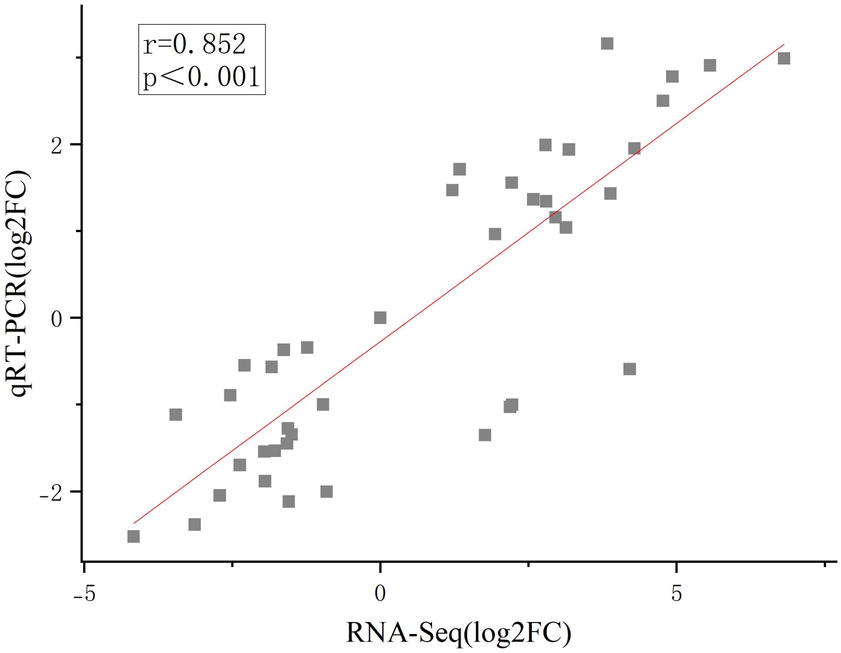

Supplementary Figure S4. Correlation between RNA-seq and qRT-PCR results. Scatter plot showing the $\log_2$FC values obtained from RNA-seq (x-axis) and qRT-PCR (y-axis) for eight selected genes across all chilling time points (n = 48). The red line represents the linear regression. Pearson correlation coefficient (r = 0.852) and P value (P < 0.01) are indicated.

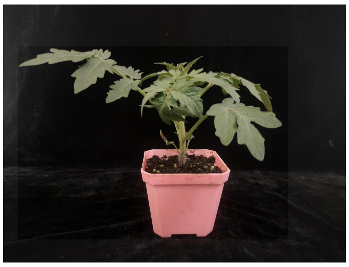

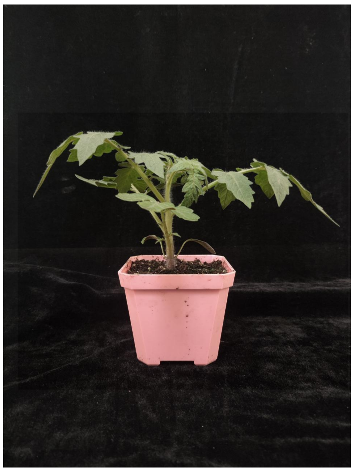

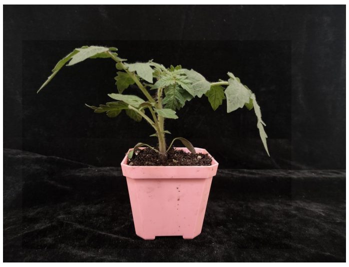

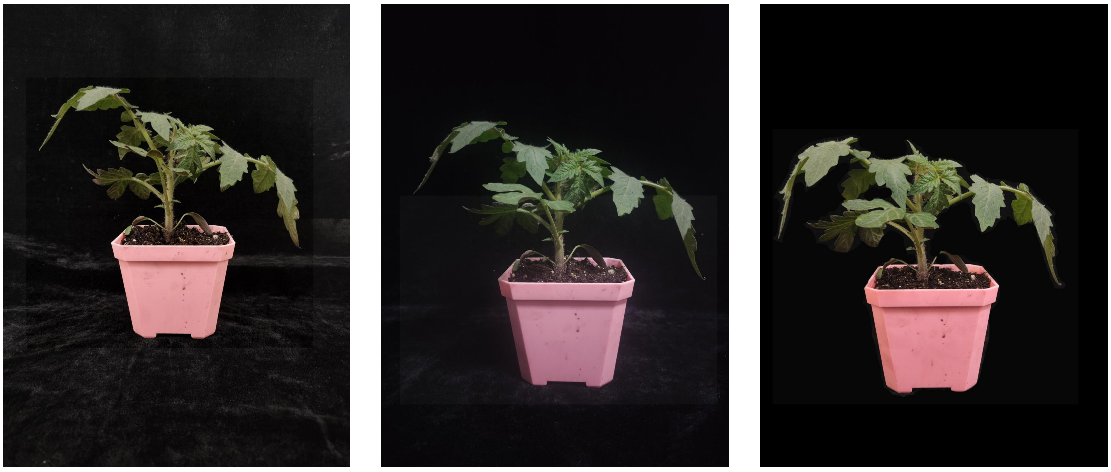

Supplementary Figure S5. Unmodified original photographs of tomato seedlings used in Figure 1. Seedlings were photographed at 0 h (CK), 1 h (DW1), 3 h (DW2), 6 h (DW3), 12 h (DW4), and 24 h (DW5) after the onset of chilling at 4 ° C. Photographs were taken with a black cloth as the original background. No background replacement, cropping of plant material, brightness/contrast adjustment, or other content modification was applied to these original images. These images are provided solely for verification of Figure 1.
